# MITF-Independent Pro-Survival Role of BRG1-Containing SWI/SNF Complex in Melanoma Cells

**DOI:** 10.1371/journal.pone.0054110

**Published:** 2013-01-17

**Authors:** Lubica Ondrušová, Jiri Vachtenheim, Jiri Réda, Petra Žáková, Kamila Benková

**Affiliations:** 1 Laboratory of Transcription and Cell Signaling, Institute of Medical Biochemistry and Laboratory Diagnostics, First Faculty of Medicine, Charles University in Prague, Prague, Czech Republic; 2 Department of Pathology, Hospital Bulovka, Prague, Czech Republic; George Mason University, United States of America

## Abstract

Metastasized malignant melanoma has a poor prognosis because of its intrinsic resistance to chemotherapy and radiotherapy. The central role in the melanoma transcriptional network has the transcription factor MITF (microphthalmia-associated transcription factor). It has been shown recently that the expression of MITF and some of its target genes require the SWI/SNF chromatin remodeling complex. Here we demonstrate that survival of melanoma cells requires functional SWI/SNF complex not only by supporting expression of MITF and its targets and but also by activating expression of prosurvival proteins not directly regulated by MITF. Microarray analysis revealed that besides the MITF-driven genes, expression of proteins like osteopontin, IGF1, TGFß2 and survivin, the factors known to be generally associated with progression of tumors and the antiapoptotic properties, were reduced in acute BRG1-depleted 501mel cells. Western blots and RT-PCR confirmed the microarray findings. These proteins have been verified to be expressed independently of MITF, because MITF depletion did not impair their expression. Because these genes are not regulated by MITF, the data suggests that loss of BRG1-based SWI/SNF complexes negatively affects survival pathways beyond the MITF cascade. Immunohistochemistry showed high expression of both BRM and BRG1 in primary melanomas. Exogenous CDK2, osteopontin, or IGF1 each alone partly relieved the block of proliferation imposed by BRG1 depletion, implicating that more factors, besides the MITF target genes, are involved in melanoma cell survival. Together these results demonstrate an essential role of SWI/SNF for the expression of MITF-dependent and MITF-independent prosurvival factors in melanoma cells and suggest that SWI/SNF may be a potential and effective target in melanoma therapy.

## Introduction

Malignant melanoma is highly invasive and early metastasizing tumor, and its incidence has been increasing in recent years [1], [2]. In the melanocyte lineage, MITF-M (melanocyte-specific isoform of MITF, referred to as MITF in the text), a member of the large basic helix-loop-helix leucine zipper family of transcription factors, plays an essential role in the embryonic development, maintenance of lineage identity, and differentiation. MITF is central for the transcription of genes involved in various cellular processes from embryonic development of melanocytes to metastasis of melanoma [3], [4], [5]. Targets of MITF include genes involved in pigment formation [5], cell cycle regulation (p21 and CDK2) [6], [7], apoptosis (Bcl-2 and livin) [8], [9] and organization of cytoskeleton (diaphanous-related formin Dia1) [10]. MITF expression is heterogeneous in advanced melanomas [11] but is highly expressed at the early phases of melanocyte transformation.

SWI/SNF chromatin remodeling complexes are consisting of about 12 proteins, and are present in cells as several subcomplexes having only subtle differences in subunit composition [12], [13], [14]. They alter the local nucleosome structure at the promoter regions to regulate transcription. These complexes use the energy provided by either BRM (Brahma, SMARCA2) or BRG1 (Brahma-related gene, SMARCA4), two homologous enzymes with ATPase activity which are present in the complexes in a mutually exclusive manner [14]. The complexes containing BRG1 or BRM may have distinct specificity toward different promoters or may function promiscuously depending on cell and promoter context.

Two subunits of the SWI/SNF complex, INI1/hSNF5/BAF47 and BRG1, are regarded as tumor suppressors. The INI1/hSNF5 subunit is a bone fide tumor suppressor whose homozygous inactivation results in rhabdoid tumors in humans [15]. A number of reports have demonstrated that BRG1 or BRM are downregulated or inactivated in cancer cell lines and tumor samples derived mostly from non-small cell lung cancer. BRG1 has been described frequently mutated in lung cancer cell lines [16]. In contrast to BRG1, BRM is inactivated by epigenetic mechanisms [17]. Loss of BRM or BRG1 was implicated in cancer progression [14], [17], [18], [19]. This was partly attributed to the necessity of BRG1 in Rb-mediated cell cycle arrest [20], [21]. However, the function of SWI/SNF is controversial because some cancer cells such as from gastric or prostate tumors have aberrantly increased expression of BRG1 [22], [23]. Thus, SWI/SNF can behave also as a tumor promoter, depending on the cancer tissue context.

Expression of MITF and several pigment cell-specific MITF target genes have been previously reported to be dependent on SWI/SNF chromatin remodeling complex [24], [25], [26]. It has been shown that expression of dominant-negative (DN) mutants of BRM and BRG1 repressed transcription of melanocyte markers (tyrosinase, Trp1, dct, and silver), the expression of which was induced by exogenous MITF in murine fibroblasts [24]. In human melanoma cell lines, many MITF targets require the SWI/SNF complex for expression. As for MITF expression, either BRG1 or BRM can be present in the complex [25], [26], [27] to activate transcription of MITF targets. Moreover, BRG1 expression was found increased in primary melanoma and metastatic melanoma when compared with dysplastic nevi and reduction of BRG1 expression by siRNA in melanoma cell lines resulted in a significantly decreased cell proliferative ability [28], concordant with our previous results [26] and findings presented here. Furthermore, BRG1 expression has been reported to be elevated during melanoma progression and modulated the expression of some of the extracellular matrix and adhesion proteins [29] such as the metastasis-associated protein metalloproteinase (MMP) 2, which contributed to the BRG1-mediated increase of melanoma invasiveness [29].

In the present work, we identify novel proteins repressed by the inactivation of BRG1 expression, which are not directly regulated by MITF. Importantly, we find here that several additional pro-survival proteins (osteopontin, IGF1, TGFß2, and survivin) are downregulated after the acute depletion of BRG1, together implicating SWI/SNF as a transcription coactivator important for survival and proliferation of melanoma cells. We also demonstrate that the BRG1 knockdown suppressed proliferation of melanoma cells and both BRG1 and BRM are highly expressed in melanoma samples. These results indicate that BRG1 is involved in melanoma progression and possibly metastasis through both the MITF-dependent and independent mechanisms and suggest the disparity of the SWI/SNF function in different types of cancer cells.

## Results

### Ablation of SWI/SNF Function by the BRG1 Knockdown in 501mel Melanoma Cells Inhibits Proliferation, Decreases Viability and Induces Apoptosis

**Figure 1:**
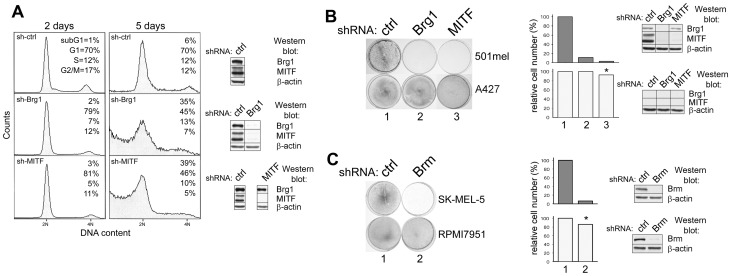
Flow cytometry and colony formation of melanoma cells after knockdown of BRG1, MITF or BRM. **A.** Effect of BRG1 or MITF depletion on DNA content profile determined by flow cytometry in 501mel cells. Cells were analyzed 2 days after selection in 2.5 µg/ml puromycin (left), or combined (detached and adherent) cells were analyzed 5 days after selection (right). All cells killed by puromycin were removed shortly after antibiotic selection and only puromycin resistant cells were assayed. Right, Western blots verifying the knockdown efficiency for BRG1 and MITF were performed after 2 days followimg the selection. **B.** BRG1 or MITF depletion inhibits colony formation in 501mel cells. A427 cells (BRG1 and BRM negative) were not affected by sh-BRG1. Middle, the quantitation of cells on dishes shown on the left. Right, Western blots verifying the knockdown efficiency for BRG1 and MITF. **C.** BRM depletion inhibits cell growth in BRG1-negative SK-MEL-5 melanoma cells, but not control RPMI7951 melanoma cells, which are BRM and BRG1-positive [26] but MITF-negative [33]. Right, Western blots showing the efficient shRNA-mediated BRM downregulation. *, not statistically significant (P<0.01).

Acute silencing of MITF causes cell cycle arrest in 501mel cells [10]. Consistently, both sh-BRG1 and sh-MITF-transfected cells revealed diminution of cell numbers in the S/G2 phases of the cell cycle after two days following puromycin selection (Figure 1A, left panels). However, when cells were analyzed after additional 3 days in culture, enhanced cell detachment was observed, accompanied by a prominent increase of cell counts in the sub-G1 phase, indicating apoptosis (Figure 1A, right panels). Apoptotic nuclei were observed in the remaining adherent cells in sh-MITF and sh-BRG1 cells but not in controls (Figure S1). In addition, detached and remaining attached cells were separately analyzed by flow cytometry and high sub-G1 and G1 contents were observed for detached cells (Figure S2) while no G2/M and very low S phase of the cell cycle were dectected, indicating that detached cells did not proliferate and most of them succcumbed to apoptosis. To further document the apoptotic process appearing 3 days after puromycin selection, we detected apoptosis by TUNEL assay (Figure S3). In this case, more prominent apoptosis was seen in sh-MITF than in sh-BRG1 cells. This might be due to a more immediate effect of MITF (which directly activates many apoptosis-inducing genes), while the apoptosis caused by silencing of BRG1 may require more time because MITF expression has to be inhibited first. No apoptosis was detected in sh-control cells. Expression of both BRG1 and MITF was effectively blocked by appropriate shRNA, as assessed by Western blots (Figure 1A, right). However, only slight activation of caspase 3 and 7 and no PARP cleavage was revealed (not shown), implying that cell death in sh-BRG1 and sh-MITF-transfected 501mel cells was predominantly caspase-independent. Similar caspase-independent triggering of apoptosis has also been observed in melanoma cells after the inactivation of survivin function by its dominant negative mutant [30].

Expression of MITF has been associated with proliferation and survival of melanoma cells via its target genes CDK2, Bcl-2, livin, c-met, and HIF1α [6], [8], [9], [31], [32]. Indeed, both BRG1 or MITF depletion severely reduced proliferation of 501mel cells, whereas the growth of BRG1/BRM non-expressing A427 lung carcinoma cells (used as a control to exclude a possible off target effects of sh-BRG1) was not inhibited, indicating that the effect of sh-BRG1 plasmid was specific (Figure 1B, upper, left). Depletion of BRM in BRG1-negative SK-MEL-5 cells [26] was sufficient to block proliferation, whereas the growth of BRG1/BRM-expressing RPMI7951 melanoma cells, which are exceptionally MITF-negative [33], was not affected (Figure 1C). Western blots (Figure 1B right) show efficient inhibition of BRG1 or MITF expression in 501mel cells, or BRM in SK-MEL-5 cells.

**Figure 2:**
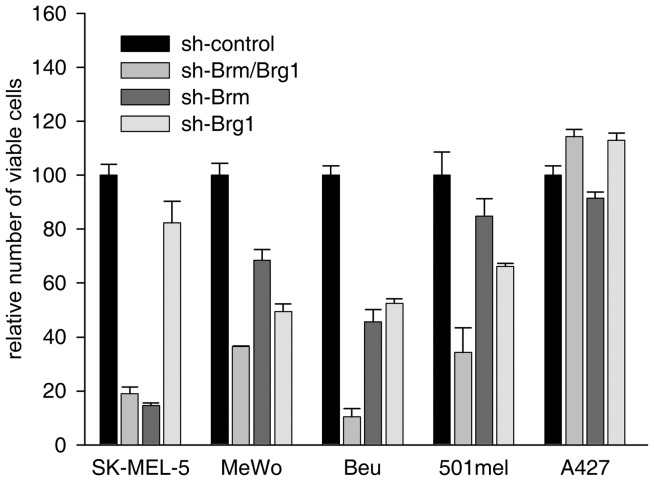
Viability of melanoma cell lines after shRNA-mediated depletion of BRG1, BRM, or both. Viability of each cell line is indicated in the histogram. The results are expressed as per cents of control shRNA-transduced cells. The cells were infected with appropriate lentivirus, replated two days later into 12-well plates, and viability was determined after next 3 days. The values are means of triplicates +-SE. One of the three independent experiments is shown. No decrease of viability was observed in BRM/BRG1-null A427 cells, used as a negative control. The knockdown of BRG1, BRM or common BRG1/BRM was efficient (see Figures 1, 3B, and 4, and Figure 3 in our previous article [26]).

Further, cell viability was negatively impacted to various degree upon knockdown of BRM, BRG1, or both, in MeWo, Beu and 501mel cells, while only BRG1-negative SK-MEL-5 cells became less viable after BRM-depletion alone (Figure 2). The highest decrease of viability in cell lines expressing both BRG1 and BRM was seen when both were depleted concomitantly by using the common BRG1/BRM shRNA. All these results are in line with the necessity that at least one ATPase must be present for proliferation and viability of each cell line [26]. Cell viability was not affected in A427 cells used as controls because they do not express either BRG1 or BRM.

### Microarray Analysis of Gene Expression after BRG1 Knockdown in 501mel Cells

**Figure 3:**
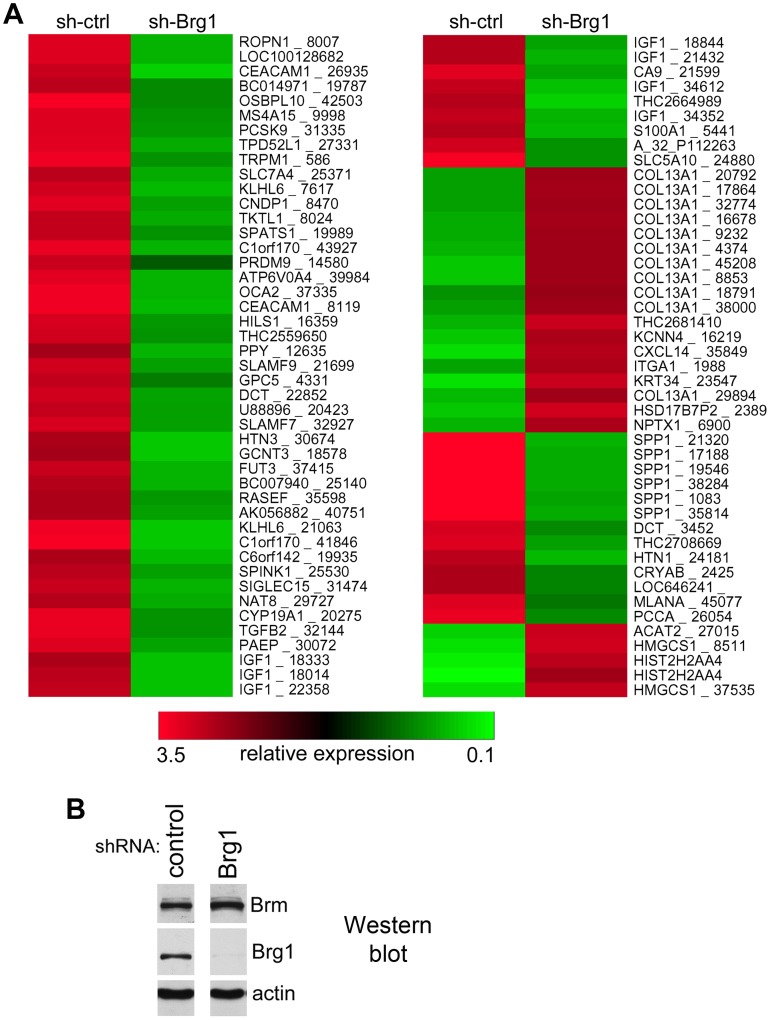
Microarrays showing transcriptional response of BRG1 knockdown in 501mel cells. **A.** Microarray analysis of genes that are differentially expressed between sh-control-transfected and sh-BRG1-transfected 501mel cells. Heat map shows only genes whose expression was changed more than 8-fold (P<0.005). Color key (bottom, the highest expression is in red color) shows the relative range of expression. Genes the expression of which was down- or up-regulated >2-fold (P<0.005) are listed in Tables S1 and S3, respectively. Control and BRG1-shRNA transfected 501mel cells were analyzed immediately after a short (2-day) selection in puromycin to prevent cell death or reexpression of MITF due to the persisting BRM [26]. Two experiments (in duplicates) were carried out with similar results and one is presented. **B.** Western blot confirming the efficiency of BRG1 knockdown in cells which were used for microarray analysis. BRM protein expression was not appreciably affected (not shown and [26]).

We next used cDNA microarrays to investigate the transcriptional response to the BRG1 depletion in a genome-wide analysis (Figure 3A). We chose the BRG1 knockdown, because its depletion resulted in much stronger inhibition of MITF and its three analyzed target genes (melastatin, CDK2, and tyrosinase) than ablation of BRM in 501mel cells, implicating that BRG1-based SWI/SNF complexes may play more important role in expression of these and possibly other target genes [26]. The changes in gene expression were studied shortly after the complete inhibition of BRG1 (2 days after a 2-day selection in puromycin), ensuring that the changes in expression profile were due exclusively to BRG1 loss. In the microarray analysis, we found that expression of 381 genes (represented by 485 probes) had been downregulated >2-fold (P<0.005) (Table S1), including some of the known MITF targets (Table S2), while other MITF targets were decreased to a lesser extent (not shown). Sox10, the upstream MITF transactivator, was also repressed, by ∼5-fold (Table S1). By contrast, a smaller number of genes (210, represented by 273 probes) was upregulated >2-fold (P<0.005) (Table S3). Interestingly, among the strongly repressed genes (67 genes, downregulated >8-fold, P<0.005; Figure 3A and Table S1) are insulin-like growth factor 1 (IGF1) and osteopontin (OPN, also named SPP1), two factors known to have anti-apoptotic, proliferative, and pro-invasive activity in melanoma [34], [35]. Notably, the expression of TGFß2, a secreted growth factor overexpressed in several tumor types including melanomas [36], was also downregulated ∼12-fold (Table S1). TGFß/SMAD pathway contributes to melanoma invasiveness [36], [37] and TGFß2 has been shown to be an important determinant of melanoma brain metastases [38]. An antiapoptotic protein survivin was downregulated ∼1.9-fold (data not shown). Of the smaller set of >8-fold upregulated genes, 10 probes (out of 23) detected collagen type XIIIα1, which was previously reported to participate in epidermal cell-cell adhesions [39]. An efficient acute suppression of BRG1 expression was achieved in cells in which microarray analysis was performed, as shown by W. blot (Figure 3B). Additionally, the cell cycle driving protein E2F1 was downregulated about ∼4-fold (Table S1) and the cdk inhibitor p15 was upregulated ∼5-fold (Table S3). These changes might have contributed to the antiproliferative phenotype after BRG1 depletion. Collectively, the microarrays confirmed the requirement of BRG1 for the maintenance of pigment cell-specific transcription regulated by MITF and, importantly, revealed a positive role of SWI/SNF for expression of several novel prosurvival molecules in melanoma cells, which are not known to be connected to the MITF axis.

### Verification of Decrease of Prosurvival Molecules in Melanoma Cells

**Figure 4:**
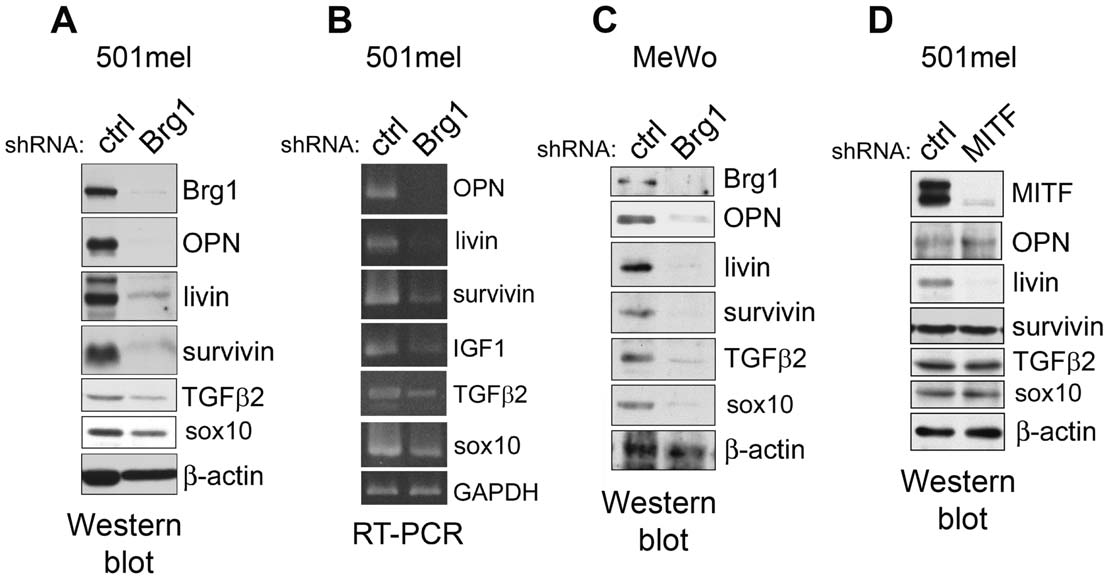
Several prosurvival molecules are decreased in BRG1-depleted cells and are not affected by sh-MITF. **A.** Decreased protein expression of OPN, livin, survivin, TGFß2 and Sox10 after BRG1 silencing in 501mel cells. **B.** Decreased mRNA levels for the same proteins after BRG1 silencing in 501mel cells. **C.** Similar decrease of expression levels for the proteins analyzed in (A) performed in MeWo cells. **D.** Verification that OPN, survivin, TGFß2 and Sox10 are not regulated by MITF since MITF depletion in 501mel cells did not have any effect on their protein levels. Livin alone was decreased because it is a MITF target. ß-actin used as a control was not changed in all experiments.

Although most of the effects caused by BRG1 knockdown on proliferation could be mediated by repression of MITF and hence its targets, several prosurvival factors (OPN, IGF1, survivin and TGFß2), the transcription of which was not reported to be MITF-responsive, were downregulated by BRG1 depletion, as revealed by microarray analysis. All of these proteins were previously implicated as pro-oncogenic factors in melanoma, promoting proliferation, antiapoptosis, invasiveness, or metastasis [30], [34], [37], [40]. To validate the microarray results, we estimated the protein expression and found that protein levels of OPN, survivin, TGFß2, and also livin, a MITF transcriptional target added as a control for MITF-driven gene, were substantially decreased in BRG1-depleted cells (Figure 4A). Sox10 (an upstream MITF regulator) was also decreased, but to a lesser extent (Figure 4A). We were not able to detect an appreciable expression level of the very small molecule IGF1 on W. blots, probably because this autocrine factor could be secreted early from cells and stimulates growth through the binding to its receptors. Nevertheless, IGF1 mRNA was lowered in 501mel cells (Figure 4B). The mRNA levels of OPN, livin, survivin, IGF1, TGFß2, and Sox10 were also significantly reduced, by RT-PCR (Figure 4B) and real-time PCR (for survivin, 5-fold, data not shown). By contrast, the mRNA levels of two antiapoptotic proteins Bcl2 and Mcl-1 remained unchanged, by real time PCR (not shown). To further verify that all these protumorigenic factors were downregulated after BRG1 depletion also in different melanoma cells, we used melanoma cell line MeWo, where we proved that the same factors were downregulated similarly as in 501mel cells (Figure 4C). Thus, these factors generally need the presence of BRG1 for their expression in melanoma cells.

### Expression of OPN, IGF1, TGFß2, and Survivin is not Affected by MITF Knockdown in Melanoma Cells

The expression of OPN, IGF1, TGFß2, and survivin was not reported previously to be MITF-dependent in melanomas. Nevertheless, we wished to exclude that the decrease of these molecules were caused by the depletion of MITF or genes downstream of its cascade. We blocked MITF in 501mel cells by shRNA and performed a Western blot of an extract from both blocked and control cells. As expected, none of the OPN, TGFß2, survivin, and Sox 10 proteins was affected by severe MITF downregulation, while expression of livin, a MITF target, almost disappeared (Figure 4D). Thus, we verified that the above cancer prosurvival factors and Sox10 require BRG1, not MITF, for expression.

### Expression of BRG1 and BRM in Melanoma Tumor Specimens and Nevi

**Figure 5:**
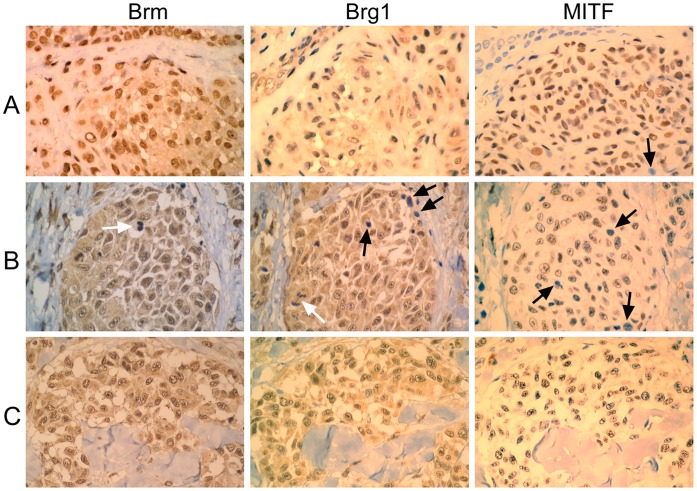
Immunostaining of BRM, BRG1, and MITF in nevi and primary melanomas. **A.** Intradermal nevus; **B** and **C.** Primary melanomas. Parallel sections (5 µm) were stained with antibodies against BRM (left), BRG1 (middle), and MITF (right). Protein expression was analyzed in intradermal or compound nevi (5 sections) and primary melanomas >1 mm in thickness (9 sections) and representative images were shown. Black arrows indicate negative interphase nuclei in BRG1 and MITF staining, open arrows mark BRM- or BRG1-negative mitotic nuclei. Scoring (0–3) is described in Materials and Methods. Magnification, ×400.

The results suggest that the presence of either BRG1 or BRM is pivotal for MITF expression [26]. We therefore asked whether cells in the tumor tissue maintain expression of at least one ATPase. Immunohistochemical analysis (Figure 5) revealed that BRM protein was highly expressed in nevi (5 analyzed samples) and tumors (9 samples of primary melanomas) (score predominantly 3); no BRM-negative cells were found. Detection of BRG1 generally showed more heterogeneous staining (score 2 or 3) with a higher proportion of moderate expression levels in nevi compared with melanomas. Also, increased number of weakly stained nuclei and very rare BRG1-negative cells (score 0) were detected in some melanoma specimens. MITF immunostaining showed heterogeneity of expression in nevi and melanomas (score 0–3) with some cells being devoid of MITF expression (Figure 5), in concert with previous findings [10]. The results together show that both ATPases are expressed in melanomas and whereas BRG1 expression might be attenuated or rarely entirely lost in some tumor cells, BRM positivity is maintained in nevi and primary melanomas.

### Partial Rescue of Cell Proliferation by CDK2, Osteopontin, and IGF1

**Figure 6:**
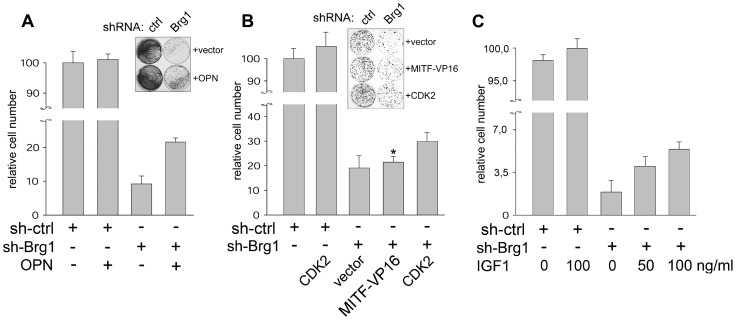
Separate restitution of OPN, CDK2 or IGF1 partly rescued cell proliferation. A. Addition of recombinant OPN (50 µg/ml) can partly increase colony formation of BRG1-silenced 501mel cells. OPN has been added 1 day after shRNA encoding plasmid and fresh OPN was replaced two days later. Cells were analyzed 5 days after transfection. The values are means of duplicates +-SE. One of four independent experiments is shown. **B.** Overexpression of CDK2 (by use of a CDK2-FLAG expression plasmid), but not the MITF-VP16 chimera [41] slightly rescues colony formation of BRG1-silenced cells 501mel cells. *, not statistically significant (P<0.05), compared to control. CDK2-FLAG was efficiently expressed, estimated by Western blot (not shown). **C.** Exogenously added recombinant IGF1 protein partly restores colony formation of BRG1-depleted 501mel cells in a dose-dependent manner. No effects of added recombinant factors or CDK plasmid were seen in control shRNA cells. The values are means of duplicates +-SE. Western blot detecting OPN and RT-PCR detecting IGF1 mRNA showed no increase of their levels in cell extract (not shown), evidently because these factors are added as recombinant proteins and exert their effects via cell receptors. In B and C, one of three independent experiments is depicted. Relative cell number was estimated by staining with crystal violet as described in Materials and Methods.

Next, we explored the consequences of overexpressing several prosurvival proteins and MITF on proliferation of BRG1-depleted cells. Addition of recombinant OPN protein alone rescued the colony formation by about ∼2-fold (Figure 6A), whereas individually overexpressed livin, survivin, or Bcl-2 failed to show any effect (data not shown). Overexpression of the MITF-Vp16 chimera, a transcriptionally more active MITF derivative [41], only insignificantly increased the number of colonies, presumably because the expression of some downstream targets involved in proliferation are also SWI/SNF-dependent [25], while the MITF target CDK2 was more effective (Figure 6B). Furthermore, the presence of IGF1 protein in the culture medium increased proliferation of BRG1-blocked cells in a dose-dependent manner up to about 3∼fold (Figure 6C). Together, although the individual effects of exogenously supplemented molecules on restoring growth were generally mild, the results suggest that mainly the decreased CDK2 and OPN levels may be partly responsible for the proliferation defect and a combined decrease of survival factors might contribute to growth inhibition and apoptosis, either directly or indirectly, elicited by blocking the SWI/SNF function in melanoma cells.

## Discussion

Although both BRG1 and BRM display extensive homology and are interchangeable in function to support MITF and its targets expression in melanoma, their function in SWI/SNF complexes may not be equivalent. For example, these ATPases were differentially required for the expression of p53-induced p21 and MDM2 [42]. Also, an opposite role of BRM- versus BRG1-containing complexes was suggested during osteoblast differentiation [43] and maintenance of chromatin integrity was specifically dependent on BRG1 [44]. Our previous results suggest that BRG1 is fully capable of maintaining MITF expression and its acute knockdown abrogate several MITF target genes. Nevertheless, BRM can also support MITF expression in 501mel cells if it is present as the only ATPase in melanoma cells [26]. A similar compensatory role of BRM has been described previously when BRM was able to restore the Rb-dependent sensitivity to cell cycle arrest [45] or coactivate the induction of p21 in BRG1-knocked down MCF7 cells [42].

Several reports have suggested that BRM may constitute an important constraint against malignant transformation. In normal melanocytes, BRM is transiently recruited into Rb- and HDAC1-containing complexes when heterochromatin has been formed during cellular senescence [46]. Loss of BRM also promoted proliferation of prostate epithelial cells [47] and was associated with poorly differentiated gastric carcinomas [48]. Expression of BRM was also found silenced in cell lines derived from multiple tumor types through an epigenetic mechanism and restoration of its expression by HDAC (histone deacetylases) inhibitors was considered as an approach in cancer therapy [17]. By contrast, we observed that BRM was expressed not only in all melanoma cell lines analyzed, but also in nevi and primary melanomas.

Becker et al. [49] recently described undetectable BRG1 protein in more than a half of primary and metastatic melanomas while BRM negativity was much less frequent. Although BRG1 negativity in their work was much more frequent than detected here, the tendency of preferential BRG1 loss with retained BRM expression was similar in both studies. Thus, BRM might become a crucial remaining ATPase maintaining the expression of MITF and perhaps additional new targets described here in melanoma cells in vivo under conditions of BRG1 loss [26], especially in later stages of tumor progression. In tumor specimens, we also observed MITF-negative cells, which were evidently BRM/BRG1-positive. It is possible that these cells survive and proliferate due to the expression of multiple pro-proliferative and antiapoptotic non-MITF-targets identified here.

Our recent silencing experiments further indicate that melanoma cells exert defects in proliferation when both BRG1 and BRM are depleted [26], which could be attributed to the loss of MITF expression. However, reexpression of hyperactive MITF-Vp16 construct is not sufficient to restore growth (Figure 6B), presumably because the expression of proliferation-related downstream targets are also SWI/SNF-dependent [25] and/or other factors besides MITF, as described here, may be important for survival. Moreover, since melanoma lines were found to be differentially sensitive to MITF depletion [50], MITF target genes might play distinct roles in survival of individual cell lines. We also observed prominent increase of collagen type XIII expression in BRG1-blocked cells, and a similar induction of this molecule involved in cell-cell contact has been described earlier in cancer cells, though stromal ones [51], and proposed to have pro-metastatic properties.

Recently, other authors described the appearance of senescent cells rather than apoptosis upon inhibition of MITF expression [52], [53]. These results are not so contradictory and could be explained by different experimental design employed by Giuliano et al. [53], who used RNA duplexes for MITF inhibition, a short pulse of transfection, and used conditioned medium in some experiments [52]. Instead, we used a plasmid producing continuously very efficient sh-BRG1. In our experiments, several days after transfection, the cells were grossly deteriorated with a large portion of detached cells and apoptosis was detected. According to the postulated rheostat model for MITF levels in melanoma cells [10], small changes of MITF level can cause very different cellular effects. We argue that in the described experiments [53] MITF could have dropped to a low level causing senescence, while sustained complete inhibition of MITF expression may indeed lead to apoptosis in 501mel cells.

Importantly, our results further indicate that the SWI/SNF complex may be crucial for the activity of MITF-independent pathways resulting in upregulation of critical factors such as OPN or IGF1. Both of these molecules were capable of rescuing proliferation of BRG1-depleted 501mel cells, albeit their overall effect was mild. Presumably, combined silencing of MITF and prosurvival growth factors participated in loss of proliferation in BRG1-depleted cells. The expression of a secreted phosphoprotein OPN, which is involved in tumor progression and metastasis, is increased in many kinds of tumors including melanomas [34] and OPN promoter was shown to be a target of activated Hedgehog pathway in melanoma [40]. The Hedgehog-GLI signaling is activated in melanoma and interference with this pathway inhibits melanoma growth and prevents recurrence and metastases in mice [54]. It remains to be investigated if SWI/SNF could activate transcriptional responses to aberrantly activated Hedgehog pathway, and whether BRG1 is involved directly in transcription from promoters of IGF1, OPN, survivin, or TGFß2. Recent search for driver and passenger mutations in melanoma on the whole-genome basis by the use of exome sequencing revealed abundant passenger mutations caused evidently by UV light exposure. Only one Brg1 nonsense mutation and no Brm mutations were found [55]. These data indicate that the Brg1 and Brm are not a subject of driver and passenger mutations and indirectly support our current results that SWI/SNF ATPases are preserved intact in melanoma cells and the SWI/SNF complex plays a prosurvival role in this cancer type. Mutations of ARID2 (BAF200), ARID1A (BAF250A) and ARID1B (BAF250B), which are also SWI/SNF components [14], were also found mutated at relatively low frequencies and ARID2 mutations were predicted to be loss of function events. However, no data about the function of ARID proteins and the functional consequence of their mutations in melanoma cells are available. Evidently, more investigation is needed to delineate the function of ARIDs in melanoma in the context of SWI/SNF. In summary, as BRG1 knockdown downregulated expression of a large number of MITF-dependent and MITF-independent prosurvival proteins, our data suggest that a tissue-aimed inactivation of the SWI/SNF complex might become an effective approach in the therapy of melanoma.

## Materials and Methods

### Ethics Statement

The samples of patients (primary melanoma lesions and benign nevi) used in this work were obtained with the patientś written informed consent. The Ethics Committee of the University Hospital Bulovka, Prague, approved the written informed consent and agreed with the analysis of samples which was performed in the present work.

### Cell Lines

501mel cells were gift from Dr. R. Halaban (Yale University, New Haven, CT) and were previously published [10], [11], [52], [53]. 501mel cells were maintained in the RPMI-1640 medium supplemented with 10% fetal bovine serum (FBS) and 293FT cells (Life Technologies, Carlsbad, CA) were maintained in the DMEM medium supplemented with FBS. A427 lung carcinoma cells (ATCC, Manassas, VA) were grown in the RPMI-1640 medium and SK-MEL-5 and MeWo melanoma cells (ATCC) were maintained in the E-MEM medium supplemented with FBS, pyruvate, and non-essential amino acids (Sigma). Beu melanoma cells were described [33]. All cell lines contained 1% penicillin and 1% streptomycin in the medium.

### Plasmids, Transfections, and Chemicals

CDK2 cDNA was expressed from the pFLAG-CMV-4 (Sigma, St. Louis, MO). The MITF-Vp16 construct was described [41]. Cells were transfected using Lipofectamine 2000 (Life Technologies) and selected in 2.0 µg/ml puromycin for indicated time intervals. Recombinant human osteopontin and IGF1 were purchased from Sigma. Other chemicals were from Sigma.

### shRNA Knockdowns and Lentiviruses

The shRNA sequences were cloned in the pSUPER.puro vector (Oligoengine, Seattle, WA). The target sequence for knockdown of human MITF was GGACAATCACAACCTGATTG. The BRM, BRG1, and the common BRM/BRG1 target sequences were described previously [56], and are as follows: BRM, GGAGGTGCTAAGACACTTATG; BRG1, GTAGCTCCGAGGTCTGATAG; common BRM/BRG1, GCTGGAGAAGCAGCAGAAG. The non-targeting sequence was GACGGTACCGCGAACGAGC. Generation of lentiviral vectors (by using pLVTHM plasmid) (Addgene, Cambridge, MA) and lentiviruses were performed as described [26]. We used virus titers that infected nearly 100 per cent of cells (as assessed by GFP positivity). Cells were infected in the presence of 8 µg/ml Polybrene for 48 hrs, harvested or replated for analyses after the time intervals as indicated.

### Immunoblotting and Immunohistochemistry

Whole cell extracts were subject to Western blot analysis. Anti-MITF antibody (D5) was from Labvision (Kalamazoo, MI), and anti-ß-actin from Sigma. Other antibodies used were from Santa Cruz Biotechnology (Santa Cruz, CA) unless otherwise indicated. Primary antibodies used were against BRG1 (G-7), osteopontin (LFMb-14), livin/ML-IAP (H-90), IGF1 (H-70), and survivin (D-8). Anti-Sox10 antibody was from Abcam (Cambridge, UK) (ab25978). Proteins were visualized using ECL (GE Life Sciences, Buckinghamshire, UK) according to the manufacturer’s instructions.

Primary melanoma lesions and benign nevi were obtained with the patientś informed consent and with the approval of the Ethics Committee of the University Hospital (see Ethics statement above). Parallel tissue sections were stained with primary antibodies against BRM (Abcam, ab15597), BRG1 (H-88, Santa Cruz) or MITF (D5, Dako, Glostrup, Denmark). The detection of antigen/antibody complexes were performed using EnVision+ avidin-biotin detection system (Dako). Because Brm and Brg1 contain high sequence similarity, the specificity of primary antibodies were verified by Western blot and immunofluorescence in Brg1+/Brm- (A549) and Brg1−/Brm+ (G401) cell lines (not shown) and antibodies were proved to be strictly specific. Score was obtained for each sample of tissue section. Sections were independently examined by two pathologists. Tissues were scored on a scale of 0 (negative) to 3 (highly positive) based on the intensity of staining.

### Reverse Transcription-PCR

Total RNA was extracted from cells using TRIZOL reagent (Life Technologies) and cDNA was synthesized from 2 µg of total RNA using the SuperScript II RNase H- reverse transcriptase (Life Technologies) with an oligo-dT primer. Detection of gene expression by RT-PCR was carried out with primers pairs purchased from Santa Cruz (for OPN, livin, survivin, IGF1, TGFß2, and Sox10). Primers for the detection of control GAPDH gene expression were 5′-TGAAGGTCGGAGTCAACGGATTTGGT (fw) and 5′-CATGTGGGCCATGAGGTCCACCAC (rev).

### Apoptosis, Colony Formation, and Viability Assays

Cells were evaluated for DNA content by flow cytometry. Cells were washed, incubated in 0.2mol/l sodium phosphate-0.1mol/l citric acid solution (pH 7.8), digested with RNase A and stained with propidium iodide before analysis in a FACSCalibur (BD Biosciences, San Jose, CA). Results were analyzed by CellQuest software. Detection of apoptosis with TUNEL assay was performed by the use of APO-DIRECT™ kit (BD Biosciences) according to manufactureŕs instructions without the propidium iodide staining. Microscopic images of apoptotic nuclei in cultured cells were taken on IX51 Olympus fluorescence microscope. Caspase 3/7 activities were detected with a kit obtained from Enzo Life Sciences (Farmingdale, NY). For colony formation assays, cells were replated two days after transfection into the puromycin-containing medium, and stained with crystal violet after 12 days. Cell viability was assessed by growing lentivirus-infected cells in 24-well plates and staining with the MTT cytotoxicity/proliferation kit (Sigma) in triplicate samples. No decrease of viability was observed in BRM/BRG1-null A427 lung carcinoma cells, which were used as a negative control (Figure 2). For cell growth quantitation, cells were fixed and stained with crystal violet and the stain was then dissolved in wells with acetic acid/ethanol solution and the relative number of cells was estimated by spectrophotometry.

### Microarrays

Agilent Whole Human Genome (4x44K) expression arrays (Agilent Technologies, Santa Clara, CA) were used according to protocols recommended by the manufacturer. Total RNA was isolated using TRIZOL reagent. RNA labeling, hybridization, scanning and data acquisition was performed at AtlasBiolabs (Berlin, Germany). Primary data were generated with Agilent´s Feature Extraction Software (version 10.5.1.1) from which differential gene expression ratio values were calculated. Data analysis was done using the R-project (v. 2.8.1, www.r-project.org) and BioConductor (v. 2.3, www.bioconductor.org) software. Adjusted P value was calculated by the method of Benjamini-Hochberg.

### Statistical Methods

Statistical analyses of proliferation rates, real-time RT-PCR, and luciferase assays were done using Student’s t test.

## Supporting Information

Figure S1 **Apoptotic nuclei in sh-MITF and sh-BRG1 treated cells.** Apoptosis was detected by DAPI staining of nuclear DNA. Mounting of the remaining attached cells was performed in Vectashield mounting medium with DAPI five days after puromycin selection and observed on Olympus IX51 Olympus fluorescence microscope. Nuclei with irregular and lobular DNA staining, condensed DNA, and fragmented DNA were scored on 100 independent cells and 9 and 11 apoptotic nuclear patterns were seen in sh-MITF and sh-BRG1 cells, respectively, while no nuclei of sh-control cells showed apoptotic signs. Two representative images are shown for each shRNA (left and right panels).
(TIF)

Figure S2 **Flow cytometry of detached and adherent cells.** We performed separate flow cytometry profiles after 5 days after a 2-day puromycin selection following transfection of appropriate shRNA, similarly as in Figure 1. High content of sub-G1 and G1 phase of the cell cycle was visible in floating cell profiles. Remaining adherent cells showed normal-like profile of DNA content. Absence of G2/M phase in floating cells indicates that these cells ceased proliferating.
(TIF)

Figure S3 **TUNEL assay for the detection of apoptosis performed on 501mel cells.** Flow cytometric measurements for sh-control, sh-BRG1 and sh-MITF transfected cells after 5 days of selection in puromycin. Two days after transfection, the medium was changed to remove puromycin-killed cells. No cells remained after puromycin selection in the sample where no plasmid was present. Pooled adherent and detached cells were analyzed. No detached cells were seen in the sh-control transfected cells. The second peak indicates the extent of apoptosis, and this was more prominent in sh-MITF cells (42%) than in sh-BRG1 cells (18%) (see Results for explanation). Apoptosis was negligible (3%) in sh-control.
(TIF)

Table S1 **Microarray analysis of genes down-regulated >2-fold (P<0.005) by BRG1 depletion in 501mel cells.**
(PDF)

Table S2 **Known MITF target genes downregulated more than 2-fold by BRG1 knockdown in 501mel cells (P<0.005).** **(MITF itself was downregulated ∼5-fold)**.
(PDF)

Table S3 **Microarray analysis of genes up-regulated >2-fold (P<0.005) by BRG1 depletion in 501mel cells.**
(PDF)

## References

[1] Chin L, Garraway LA, Fisher DE (2006) Malignant melanoma: genetics and therapeutics in the genomic era. Genes Dev 20: 2149–2182. 16912270 10.1101/gad.1437206

[2] Miller AJ, Mihm MC Jr (2006) Melanoma. N Engl J Med 355: 51–65. 16822996 10.1056/NEJMra052166

[3] Goding CR (2000) Mitf from neural crest to melanoma: signal transduction and transcription in the melanocyte lineage. Genes Dev 14: 1712–1728. 10898786

[4] Levy C, Khaled M, Fisher DE (2006) MITF: master regulator of melanocyte development and melanoma oncogene. Trends Mol Med 12: 406–414. 16899407 10.1016/j.molmed.2006.07.008

[5] Vachtenheim J, Borovansky J (2010) “Transcription physiology” of pigment formation in melanocytes: central role of MITF. Exp Dermatol 19: 617–627. 20201954 10.1111/j.1600-0625.2009.01053.x

[6] Du J, Widlund HR, Horstmann MA, Ramaswamy S, Ross K, et al (2004) Critical role of CDK2 for melanoma growth linked to its melanocyte-specific transcriptional regulation by MITF. Cancer Cell 6: 565–576. 15607961 10.1016/j.ccr.2004.10.014

[7] Carreira S, Goodall J, Aksan I, La Rocca SA, Galibert MD, et al (2005) Mitf cooperates with Rb1 and activates p21Cip1 expression to regulate cell cycle progression. Nature 433: 764–769. 15716956 10.1038/nature03269

[8] McGill GG, Horstmann M, Widlund HR, Du J, Motyckova G, et al (2002) Bcl2 regulation by the melanocyte master regulator Mitf modulates lineage survival and melanoma cell viability. Cell 109: 707–718. 12086670 10.1016/s0092-8674(02)00762-6

[9] Dynek JN, Chan SM, Liu J, Zha J, Fairbrother WJ, et al (2008) Microphthalmia-associated transcription factor is a critical transcriptional regulator of melanoma inhibitor of apoptosis in melanomas. Cancer Res 68: 3124–3132. 18451137 10.1158/0008-5472.CAN-07-6622

[10] Carreira S, Goodall J, Denat L, Rodriguez M, Nuciforo P, et al (2006) Mitf regulation of Dia1 controls melanoma proliferation and invasiveness. Genes Dev 20: 3426–3439. 17182868 10.1101/gad.406406 PMC1698449

[11] Goodall J, Carreira S, Denat L, Kobi D, Davidson I, et al (2008) Brn-2 represses microphthalmia-associated transcription factor expression and marks a distinct subpopulation of microphthalmia-associated transcription factor-negative melanoma cells. Cancer Res 68: 7788–7794. 18829533 10.1158/0008-5472.CAN-08-1053

[12] Roberts CW, Orkin SH (2004) The SWI/SNF complex–chromatin and cancer. Nat Rev Cancer 4: 133–142. 14964309 10.1038/nrc1273

[13] Halliday GM, Bock VL, Moloney FJ, Lyons JG (2009) SWI/SNF: a chromatin-remodelling complex with a role in carcinogenesis. Int J Biochem Cell Biol 41: 725–728. 18723114 10.1016/j.biocel.2008.04.026

[14] Reisman D, Glaros S, Thompson EA (2009) The SWI/SNF complex and cancer. Oncogene 28: 1653–1668. 19234488 10.1038/onc.2009.4

[15] Versteege I, Sevenet N, Lange J, Rousseau-Merck MF, Ambros P, et al (1998) Truncating mutations of hSNF5/INI1 in aggressive paediatric cancer. Nature 394: 203–206. 9671307 10.1038/28212

[16] Medina PP, Romero OA, Kohno T, Montuenga LM, Pio R, et al (2008) Frequent BRG1/SMARCA4-inactivating mutations in human lung cancer cell lines. Hum Mutat 29: 617–622. 18386774 10.1002/humu.20730

[17] Glaros S, Cirrincione GM, Muchardt C, Kleer CG, Michael CW, et al (2007) The reversible epigenetic silencing of BRM: implications for clinical targeted therapy. Oncogene 26: 7058–7066. 17546055 10.1038/sj.onc.1210514

[18] Reisman DN, Sciarrotta J, Wang W, Funkhouser WK, Weissman BE (2003) Loss of BRG1/BRM in human lung cancer cell lines and primary lung cancers: correlation with poor prognosis. Cancer Res 63: 560–566. 12566296

[19] Fukuoka J, Fujii T, Shih JH, Dracheva T, Meerzaman D, et al (2004) Chromatin remodeling factors and BRM/BRG1 expression as prognostic indicators in non-small cell lung cancer. Clin Cancer Res 10: 4314–4324. 15240517 10.1158/1078-0432.CCR-03-0489

[20] Dunaief JL, Strober BE, Guha S, Khavari PA, Alin K, et al (1994) The retinoblastoma protein and BRG1 form a complex and cooperate to induce cell cycle arrest. Cell 79: 119–130. 7923370 10.1016/0092-8674(94)90405-7

[21] Bartlett C, Orvis TJ, Rosson GS, Weissman BE (2011) BRG1 mutations found in human cancer cell lines inactivate Rb-mediated cell-cycle arrest. J Cell Physiol 226: 1989–1997. 21520050 10.1002/jcp.22533 PMC4433745

[22] Sentani K, Oue N, Kondo H, Kuraoka K, Motoshita J, et al (2001) Increased expression but not genetic alteration of BRG1, a component of the SWI/SNF complex, is associated with the advanced stage of human gastric carcinomas. Pathobiology 69: 315–320. 12324708 10.1159/000064638

[23] Sun A, Tawfik O, Gayed B, Thrasher JB, Hoestje S, et al (2007) Aberrant expression of SWI/SNF catalytic subunits BRG1/BRM is associated with tumor development and increased invasiveness in prostate cancers. Prostate 67: 203–213. 17075831 10.1002/pros.20521

[24] de la Serna I, Ohkawa Y, Higashi C, Dutta C, Osias J, et al (2006) The microphthalmia-associated transcription factor requires SWI/SNF enzymes to activate melanocyte-specific genes. J Biol Chem 281: 20233–20241. 16648630 10.1074/jbc.M512052200

[25] Keenen B, Qi H, Saladi SV, Yeung M, de lS I (2010) Heterogeneous SWI/SNF chromatin remodeling complexes promote expression of microphthalmia-associated transcription factor target genes in melanoma. Oncogene 29: 81–92. 19784067 10.1038/onc.2009.304 PMC2803337

[26] Vachtenheim J, Ondrusova L, Borovansky J (2010) SWI/SNF chromatin remodeling complex is critical for the expression of microphthalmia-associated transcription factor in melanoma cells. Biochem Biophys Res Commun 392: 454–459. 20083088 10.1016/j.bbrc.2010.01.048

[27] Saladi SV, Marathe H, de lS I (2010) SWItching on the transcriptional circuitry in melanoma. Epigenetics 5: 469–475. 20543574 10.4161/epi.5.6.12315 PMC3230549

[28] Lin H, Wong RP, Martinka M, Li G (2010) BRG1 expression is increased in human cutaneous melanoma. Br J Dermatol 163: 502–510. 20491765 10.1111/j.1365-2133.2010.09851.x

[29] Saladi SV, Keenen B, Marathe HG, Qi H, Chin KV, et al (2010) Modulation of extracellular matrix/adhesion molecule expression by BRG1 is associated with increased melanoma invasiveness. Mol Cancer 9: 280. 20969766 10.1186/1476-4598-9-280 PMC3098014

[30] Liu T, Brouha B, Grossman D (2004) Rapid induction of mitochondrial events and caspase-independent apoptosis in Survivin-targeted melanoma cells. Oncogene 23: 39–48. 14712209 10.1038/sj.onc.1206978 PMC2292403

[31] Busca R, Berra E, Gaggioli C, Khaled M, Bille K, et al (2005) Hypoxia-inducible factor 1{alpha} is a new target of microphthalmia-associated transcription factor (MITF) in melanoma cells. J Cell Biol 170: 49–59. 15983061 10.1083/jcb.200501067 PMC2171372

[32] McGill GG, Haq R, Nishimura EK, Fisher DE (2006) c-Met expression is regulated by Mitf in the melanocyte lineage. J Biol Chem 281: 10365–10373. 16455654 10.1074/jbc.M513094200

[33] Vachtenheim J, Novotna H, Ghanem G (2001) Transcriptional repression of the microphthalmia gene in melanoma cells correlates with the unresponsiveness of target genes to ectopic microphthalmia-associated transcription factor. J Invest Dermatol 117: 1505–1511. 11886515 10.1046/j.0022-202x.2001.01563.x

[34] Zhou Y, Dai DL, Martinka M, Su M, Zhang Y, et al (2005) Osteopontin expression correlates with melanoma invasion. J Invest Dermatol 124: 1044–1052. 15854047 10.1111/j.0022-202X.2005.23680.x

[35] Hilmi C, Larribere L, Giuliano S, Bille K, Ortonne JP, et al (2008) IGF1 promotes resistance to apoptosis in melanoma cells through an increased expression of BCL2, BCL-X(L), and survivin. J Invest Dermatol 128: 1499–1505. 18079751 10.1038/sj.jid.5701185

[36] Javelaud D, Alexaki VI, Mauviel A (2008) Transforming growth factor-beta in cutaneous melanoma. Pigment Cell Melanoma Res 21: 123–132. 18426405 10.1111/j.1755-148X.2008.00450.x

[37] Pierrat MJ, Marsaud V, Mauviel A, Javelaud D (2012) Expression of Microphtalmia-Associated Transcription Factor (MITF), which is Critical for Melanoma Progression, is Inhibited by both Transcription Factor GLI2 and Transforming Growth Factor-beta. J Biol Chem. 10.1074/jbc.M112.358341 PMC3365743 22496449

[38] Zhang C, Zhang F, Tsan R, Fidler IJ (2009) Transforming growth factor-beta2 is a molecular determinant for site-specific melanoma metastasis in the brain. Cancer Res 69: 828–835. 19141644 10.1158/0008-5472.CAN-08-2588 PMC2633423

[39] Peltonen S, Hentula M, Hagg P, Yla-Outinen H, Tuukkanen J, et al (1999) A novel component of epidermal cell-matrix and cell-cell contacts: transmembrane protein type XIII collagen. J Invest Dermatol 113: 635–642. 10504453 10.1046/j.1523-1747.1999.00736.x

[40] Das S, Harris LG, Metge BJ, Liu S, Riker AI, et al.. (2009) The hedgehog pathway transcription factor, GLI1 promotes malignant behavior of cancer cells by upregulating osteopontin. J Biol Chem. 10.1074/jbc.M109.021949 PMC2755696 19556240

[41] Vachtenheim J, Drdova B (2004) A dominant negative mutant of microphthalmia transcription factor (MITF) lacking two transactivation domains suppresses transcription mediated by wild type MITF and a hyperactive MITF derivative. Pigment Cell Res 17: 43–50. 14717844 10.1046/j.1600-0749.2003.00108.x

[42] Xu Y, Zhang J, Chen X (2007) The activity of p53 is differentially regulated by Brm- and Brg1-containing SWI/SNF chromatin remodeling complexes. J Biol Chem 282: 37429–37435. 17938176 10.1074/jbc.M706039200

[43] Flowers S, Nagl NG Jr, Beck GR Jr, Moran E (2009) Antagonistic roles for BRM and BRG1 SWI/SNF complexes in differentiation. J Biol Chem 284: 10067–10075. 19144648 10.1074/jbc.M808782200 PMC2665061

[44] Bourgo RJ, Siddiqui H, Fox S, Solomon D, Sansam CG, et al (2009) SWI/SNF deficiency results in aberrant chromatin organization, mitotic failure, and diminished proliferative capacity. Mol Biol Cell 20 3192–3199. 19458193 10.1091/mbc.E08-12-1224 PMC2710832

[45] Strobeck MW, Reisman DN, Gunawardena RW, Betz BL, Angus SP, et al (2002) Compensation of BRG-1 function by Brm: insight into the role of the core SWI-SNF subunits in retinoblastoma tumor suppressor signaling. J Biol Chem 277: 4782–4789. 11719516 10.1074/jbc.M109532200

[46] Bandyopadhyay D, Curry JL, Lin Q, Richards HW, Chen D, et al (2007) Dynamic assembly of chromatin complexes during cellular senescence: implications for the growth arrest of human melanocytic nevi. Aging Cell 6: 577–591. 17578512 10.1111/j.1474-9726.2007.00308.x PMC1974778

[47] Shen H, Powers N, Saini N, Comstock CE, Sharma A, et al (2008) The SWI/SNF ATPase Brm is a gatekeeper of proliferative control in prostate cancer. Cancer Res 68: 10154–10162. 19074882 10.1158/0008-5472.CAN-08-1794 PMC2858924

[48] Yamamichi N, Inada K, Ichinose M, Yamamichi-Nishina M, Mizutani T, et al (2007) Frequent loss of Brm expression in gastric cancer correlates with histologic features and differentiation state. Cancer Res 67: 10727–10735. 18006815 10.1158/0008-5472.CAN-07-2601

[49] Becker TM, Haferkamp S, Dijkstra MK, Scurr LL, Frausto M, et al (2009) The chromatin remodelling factor BRG1 is a novel binding partner of the tumor suppressor p16INK4a. Mol Cancer 8: 4. 19149898 10.1186/1476-4598-8-4 PMC2644676

[50] Kido K, Sumimoto H, Asada S, Okada SM, Yaguchi T, et al (2009) Simultaneous suppression of MITF and BRAF V600E enhanced inhibition of melanoma cell proliferation. Cancer Sci 100: 1863–1869. 19659611 10.1111/j.1349-7006.2009.01266.x PMC11158511

[51] Vaisanen T, Vaisanen MR, Autio-Harmainen H, Pihlajaniemi T (2005) Type XIII collagen expression is induced during malignant transformation in various epithelial and mesenchymal tumours. J Pathol 207: 324–335. 16110459 10.1002/path.1836

[52] Ohanna M, Giuliano S, Bonet C, Imbert V, Hofman V, et al (2011) Senescent cells develop a PARP-1 and nuclear factor-{kappa}B-associated secretome (PNAS). Genes Dev 25: 1245–1261. 21646373 10.1101/gad.625811 PMC3127427

[53] Giuliano S, Cheli Y, Ohanna M, Bonet C, Beuret L, et al (2010) Microphthalmia-associated transcription factor controls the DNA damage response and a lineage-specific senescence program in melanomas. Cancer Res 70: 3813–3822. 20388797 10.1158/0008-5472.CAN-09-2913

[54] Stecca B, Mas C, Clement V, Zbinden M, Correa R, et al (2007) Melanomas require HEDGEHOG-GLI signaling regulated by interactions between GLI1 and the RAS-MEK/AKT pathways. Proc Natl Acad Sci U S A 104: 5895–5900. 17392427 10.1073/pnas.0700776104 PMC1838820

[55] Hodis E, Watson IR, Kryukov GV, Arold ST, Imielinski M, et al (2012) A landscape of driver mutations in melanoma. Cell 150: 251–263. 22817889 10.1016/j.cell.2012.06.024 PMC3600117

[56] Watanabe H, Mizutani T, Haraguchi T, Yamamichi N, Minoguchi S, et al (2006) SWI/SNF complex is essential for NRSF-mediated suppression of neuronal genes in human nonsmall cell lung carcinoma cell lines. Oncogene 25: 470–479. 16247481 10.1038/sj.onc.1209068

