# Supplementary figures and images for: MITF-Independent Pro-Survival Role of BRG1-Containing SWI/SNF Complex in Melanoma Cells
Source: PLoS One. 2013 Jan 17;8(1):e54110. doi: 10.1371/journal.pone.0054110 (PMC3547967; doi:10.1371/journal.pone.0054110)

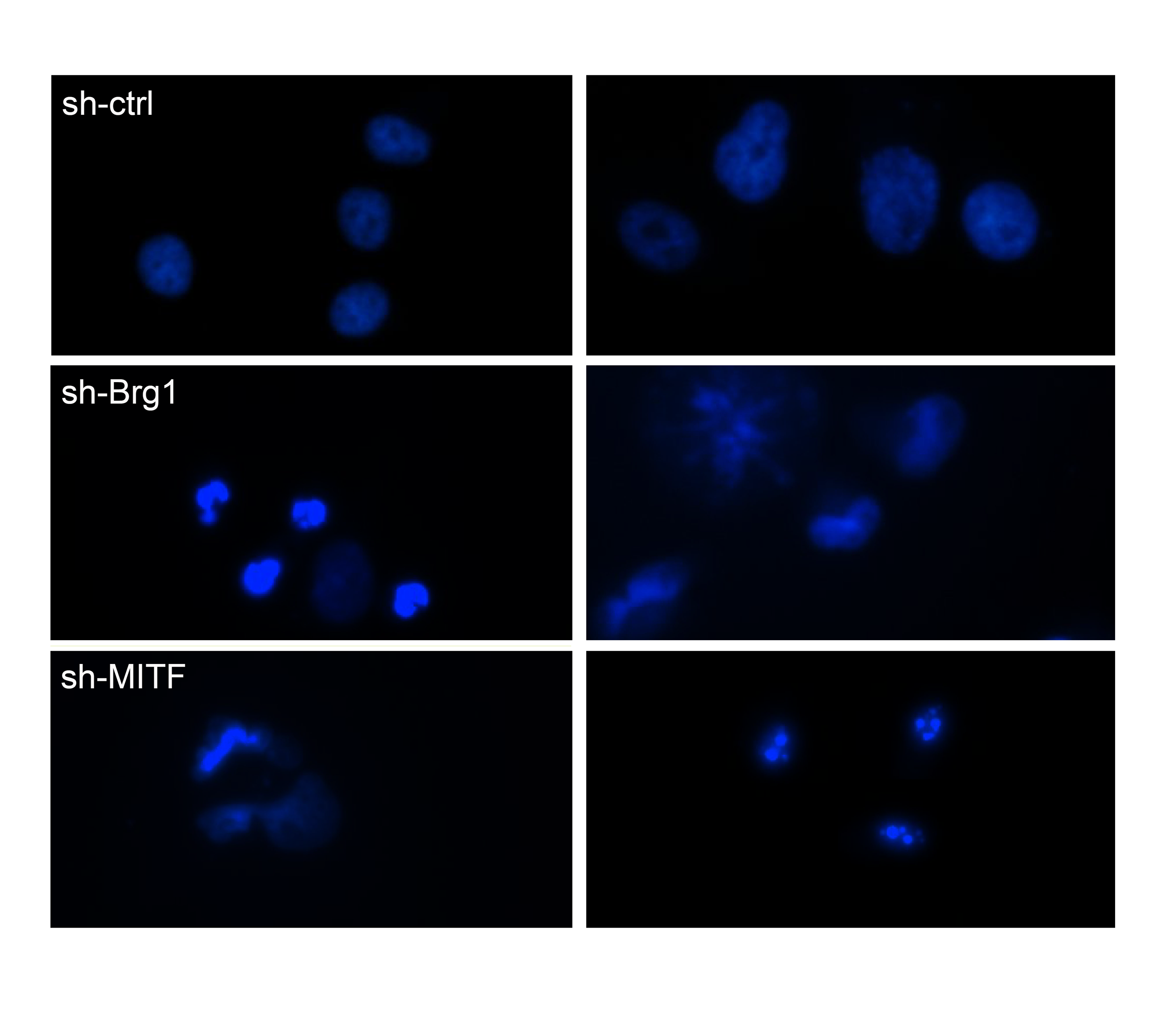

Supplement: Figure S1 — Apoptotic nuclei in sh-MITF and sh-BRG1 treated cells. Apoptosis was detected by DAPI staining of nuclear DNA. Mounting of the remaining attached cells was performed in Vectashield mounting medium with DAPI five days after puromycin selection and observed on Olympus IX51 Olympus fluorescence microscope. Nuclei with irregular and lobular DNA staining, condensed DNA, and fragmented DNA were scored on 100 independent cells and 9 and 11 apoptotic nuclear patterns were seen in sh-MITF and sh-BRG1 cells, respectively, while no nuclei of sh-control cells showed apoptotic signs. Two representative images are shown for each shRNA (left and right panels). (TIF) [file pone.0054110.s001.tif]

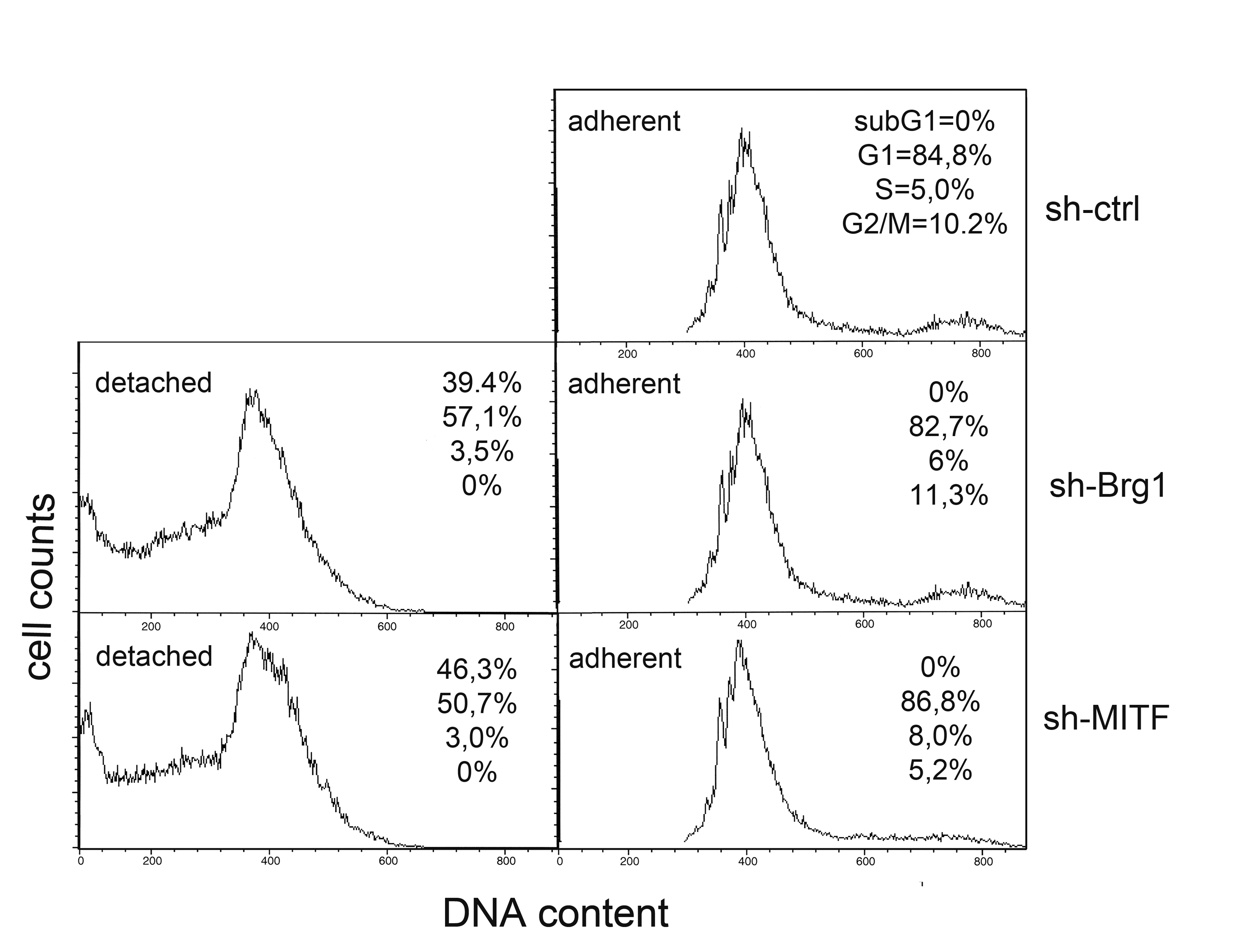

Supplement: Figure S2 — Flow cytometry of detached and adherent cells. We performed separate flow cytometry profiles after 5 days after a 2-day puromycin selection following transfection of appropriate shRNA, similarly as in Figure 1. High content of sub-G1 and G1 phase of the cell cycle was visible in floating cell profiles. Remaining adherent cells showed normal-like profile of DNA content. Absence of G2/M phase in floating cells indicates that these cells ceased proliferating. (TIF) [file pone.0054110.s002.tif]

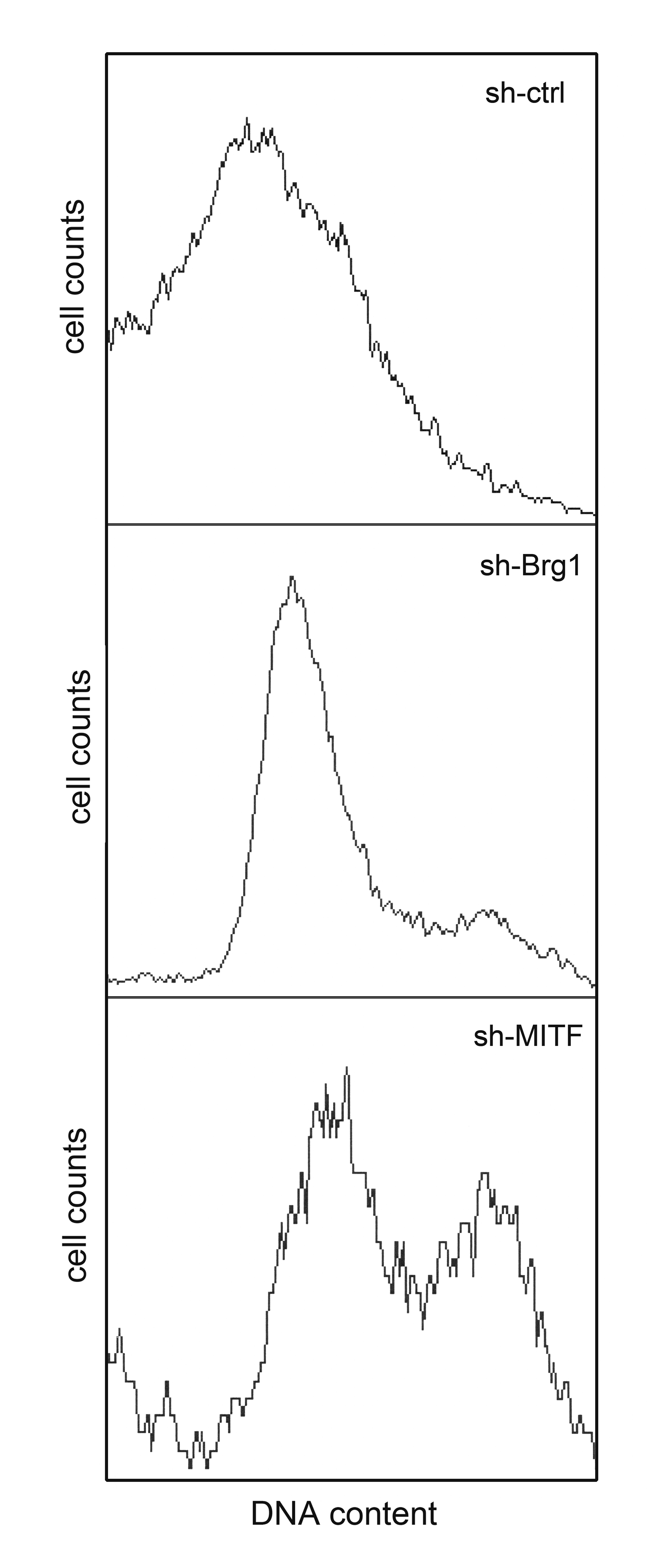

Supplement: Figure S3 — TUNEL assay for the detection of apoptosis performed on 501mel cells. Flow cytometric measurements for sh-control, sh-BRG1 and sh-MITF transfected cells after 5 days of selection in puromycin. Two days after transfection, the medium was changed to remove puromycin-killed cells. No cells remained after puromycin selection in the sample where no plasmid was present. Pooled adherent and detached cells were analyzed. No detached cells were seen in the sh-control transfected cells. The second peak indicates the extent of apoptosis, and this was more prominent in sh-MITF cells (42%) than in sh-BRG1 cells (18%) (see Results for explanation). Apoptosis was negligible (3%) in sh-control. (TIF) [file pone.0054110.s003.tif]
