# Supplementary material for: MITF-Independent Pro-Survival Role of BRG1-Containing SWI/SNF Complex in Melanoma Cells
Source: PLoS One. 2013 Jan 17;8(1):e54110. doi: 10.1371/journal.pone.0054110 (PMC3547967; doi:10.1371/journal.pone.0054110)
Supplement: Table S1 — Microarray analysis of genes down-regulated >2-fold (P<0.005) by BRG1 depletion in 501mel cells. (PDF) [file pone.0054110.s004.pdf]

Supplementary Table S1. Microarray analysis of genes down-regulated >2-fold (P<0.005) by Brg1 depletion in 501mel cells.

Gene Symbol	Systematic Name	Gene Title	Fold change
SLAMF7	NM_021181	ref|Homo sapiens SLAM family member 7 (SLAMF7), mRNA [NM_021181]	34,23
HTN1	NM_002159	ref|Homo sapiens histatin 1 (HTN1), mRNA [NM_002159]	23,30
DCT	NM_001922	ref|Homo sapiens dopachrome tautomerase (dopachrome delta-isomerase, tyrosine-related protein 2) (DCT), transcript variant 1, mRNA [NM_001922]	22,50
U88896	U88896	gb|Human endogenous retrovirus H protease/integrase-derived ORF1, ORF2, and putative envelope protein mRNA, complete cds. [U88896]	20,49
DCT	NM_001922	ref|Homo sapiens dopachrome tautomerase (dopachrome delta-isomerase, tyrosine-related protein 2) (DCT), transcript variant 1, mRNA [NM_001922]	19,45
GPC5	NM_004466	ref|Homo sapiens glypican 5 (GPC5), mRNA [NM_004466]	19,44
PAEP	NM_002571	ref|Homo sapiens progestagen-associated endometrial protein (PAEP), transcript variant 2, mRNA [NM_002571]	18,93
PCSK9	NM_174936	ref|Homo sapiens proprotein convertase subtilisin/kexin type 9 (PCSK9), mRNA [NM_174936]	18,43
PPY	NM_002722	ref|Homo sapiens pancreatic polypeptide (PPY), mRNA [NM_002722]	17,89
MS4A15	NM_152717	ref|Homo sapiens membrane-spanning 4-domains, subfamily A, member 15 (MS4A15), transcript variant 2, mRNA [NM_152717]	17,39
TPD52L1	NM_001003395	ref|Homo sapiens tumor protein D52-like 1 (TPD52L1), transcript variant 2, mRNA [NM_001003395]	16,78
TRPM1	NM_002420	ref|Homo sapiens transient receptor potential cation channel, subfamily M, member 1 (TRPM1), mRNA [NM_002420]	15,98
SLAMF9	NM_033438	ref|Homo sapiens SLAM family member 9 (SLAMF9), mRNA [NM_033438]	15,86
THC2708669	THC2708669	thc|Q3IE62_PSEHT (Q3IE62) Dihydropteroate synthase (DHPS) (Dihydropteroate pyrophosphorylase) , partial (5%) [THC2708669]	13,99
SLC5A10	NM_152351	ref|Homo sapiens solute carrier family 5 (sodium/glucose cotransporter), member 10 (SLC5A10), transcript variant 1, mRNA [NM_152351]	13,48
CEACAM1	NM_001712	ref|Homo sapiens carcinoembryonic antigen-related cell adhesion molecule 1 (biliary glycoprotein) (CEACAM1), transcript variant 1, mRNA [NM_001712]	13,14
ROPN1	NM_017578	ref|Homo sapiens ropporin, rhophilin associated protein 1 (ROPN1), mRNA [NM_017578]	12,09
TGFB2	NM_001135599	ref|Homo sapiens transforming growth factor, beta 2 (TGFB2), transcript variant 1, mRNA [NM_001135599]	12,04
TKTL1	NM_012253	ref|Homo sapiens transketolase-like 1 (TKTL1), mRNA [NM_012253]	11,97
LOC100128682	AK128099	gb|Homo sapiens cDNA FLJ46220 fis, clone TESTI4013774. [AK128099]	11,43
PRDM9	NM_020227	ref|Homo sapiens PR domain containing 9 (PRDM9), mRNA [NM_020227]	11,26
CEACAM1	NM_001024912	ref|Homo sapiens carcinoembryonic antigen-related cell adhesion molecule 1 (biliary glycoprotein) (CEACAM1), transcript variant 2, mRNA [NM_001024912]	11,26
FUT3	NM_000149	ref|Homo sapiens fucosyltransferase 3 (galactoside 3(4)-L-fucosyltransferase, Lewis blood group) (FUT3), transcript variant 1, mRNA [NM_000149]	11,12
BC007940	BC007940	gb|Homo sapiens cDNA clone IMAGE:4131853, **** WARNING: chimeric clone ****. [BC007940]	11,03
CRYAB	NM_001885	ref|Homo sapiens crystallin, alpha B (CRYAB), mRNA [NM_001885]	10,80
MLANA	NM_005511	ref|Homo sapiens melan-A (MLANA), mRNA [NM_005511]	10,76
PCCA	NM_000282	ref|Homo sapiens propionyl Coenzyme A carboxylase, alpha polypeptide (PCCA), nuclear gene encoding mitochondrial protein, transcript variant 1, mRNA [NM_	10,69
LOC646241	BC028204	gb|Homo sapiens cDNA clone IMAGE:5201079. [BC028204]	10,41
C1orf170	AK123855	gb|Homo sapiens cDNA FLJ41861 fis, clone NTONG2008672. [AK123855]	10,37
CNDP1	NM_032649	ref|Homo sapiens carnosine dipeptidase 1 (metallopeptidase M20 family) (CNDP1), mRNA [NM_032649]	10,28
SPINK1	NM_003122	ref|Homo sapiens serine peptidase inhibitor, Kazal type 1 (SPINK1), mRNA [NM_003122]	10,19
SLC7A4	NM_004173	ref|Homo sapiens solute carrier family 7 (cationic amino acid transporter, y+ system), member 4 (SLC7A4), mRNA [NM_004173]	10,17
GCNT3	ENST00000267857	ens|Beta-1,3-galactosyl-O-glycosyl-glycoprotein beta-1,6-N-acetylglucosaminyltransferase 3 (EC 2.4.1.102)(EC 2.4.1.150)(Core 2/core 4 beta-1,6-N-acetylglucos	9,93
ATP6V0A4	NM_020632	ref|Homo sapiens ATPase, H+ transporting, lysosomal V0 subunit a4 (ATP6V0A4), transcript variant 1, mRNA [NM_020632]	9,91
C1orf170	AK123855	gb|Homo sapiens cDNA FLJ41861 fis, clone NTONG2008672. [AK123855]	9,77
C6orf142	NM_138569	ref|Homo sapiens chromosome 6 open reading frame 142 (C6orf142), mRNA [NM_138569]	9,71
S100A1	NM_006271	ref|Homo sapiens S100 calcium binding protein A1 (S100A1), mRNA [NM_006271]	9,65
HILS1	NR_024193	ref|Homo sapiens histone linker H1 domain, spermatid-specific 1 (HILS1), transcript variant 1, non-coding RNA [NR_024193]	9,62
SPATS1	NM_145026	ref|Homo sapiens spermatogenesis associated, serine-rich 1 (SPATS1), mRNA [NM_145026]	9,47
IGF1	NM_000618	ref|Homo sapiens insulin-like growth factor 1 (somatomedin C) (IGF1), transcript variant 4, mRNA [NM_000618]	9,41
CYP19A1	NM_031226	ref|Homo sapiens cytochrome P450, family 19, subfamily A, polypeptide 1 (CYP19A1), transcript variant 2, mRNA [NM_031226]	9,29
IGF1	NM_000618	ref|Homo sapiens insulin-like growth factor 1 (somatomedin C) (IGF1), transcript variant 4, mRNA [NM_000618]	9,23
OSBPL10	NM_017784	ref|Homo sapiens oxysterol binding protein-like 10 (OSBPL10), mRNA [NM_017784]	9,19
IGF1	NM_000618	ref|Homo sapiens insulin-like growth factor 1 (somatomedin C) (IGF1), transcript variant 4, mRNA [NM_000618]	9,17
RASEF	NM_152573	ref|Homo sapiens RAS and EF-hand domain containing (RASEF), mRNA [NM_152573]	8,96
BC014971	BC014971	gb|Homo sapiens, Similar to tubulin, beta, 2, clone IMAGE:4873024, mRNA. [BC014971]	8,94
CA9	NM_001216	ref|Homo sapiens carbonic anhydrase IX (CA9), mRNA [NM_001216]	8,84
SPP1	NM_001040058	ref|Homo sapiens secreted phosphoprotein 1 (SPP1), transcript variant 1, mRNA [NM_001040058]	8,54
KLHL6	NM_130446	ref|Homo sapiens kelch-like 6 (Drosophila) (KLHL6), mRNA [NM_130446]	8,51
OCA2	NM_000275	ref|Homo sapiens oculocutaneous albinism II (OCA2), mRNA [NM_000275]	8,43
SIGLEC15	NM_213602	ref|Homo sapiens sialic acid binding Ig-like lectin 15 (SIGLEC15), mRNA [NM_213602]	8,41
IGF1	NM_000618	ref|Homo sapiens insulin-like growth factor 1 (somatomedin C) (IGF1), transcript variant 4, mRNA [NM_000618]	8,38
IGF1	NM_000618	ref|Homo sapiens insulin-like growth factor 1 (somatomedin C) (IGF1), transcript variant 4, mRNA [NM_000618]	8,35
KLHL6	NM_130446	ref|Homo sapiens kelch-like 6 (Drosophila) (KLHL6), mRNA [NM_130446]	8,34
SPP1	NM_001040058	ref|Homo sapiens secreted phosphoprotein 1 (SPP1), transcript variant 1, mRNA [NM_001040058]	8,32
THC2664989	THC2664989	thc|Q40J89_EHRCH (Q40J89) Cation efflux protein, partial (6%) [THC2733597]	8,32
SPP1	NM_001040058	ref|Homo sapiens secreted phosphoprotein 1 (SPP1), transcript variant 1, mRNA [NM_001040058]	8,26
AK056882	AK056882	gb|Homo sapiens cDNA FLJ32320 fis, clone PROST2003537. [AK056882]	8,25
IGF1	NM_000618	ref|Homo sapiens insulin-like growth factor 1 (somatomedin C) (IGF1), transcript variant 4, mRNA [NM_000618]	8,21
THC2559650	THC2559650	thc|ALU5_HUMAN (P39192) Alu subfamily SC sequence contamination warning entry, partial (11%) [THC2559650]	8,15
SPP1	NM_001040058	ref|Homo sapiens secreted phosphoprotein 1 (SPP1), transcript variant 1, mRNA [NM_001040058]	8,14
SPP1	NM_001040058	ref|Homo sapiens secreted phosphoprotein 1 (SPP1), transcript variant 1, mRNA [NM_001040058]	8,11
NAT8	NM_003960	ref|Homo sapiens N-acetyltransferase 8 (GCN5-related, putative) (NAT8), mRNA [NM_003960]	8,08
SPP1	NM_001040058	ref|Homo sapiens secreted phosphoprotein 1 (SPP1), transcript variant 1, mRNA [NM_001040058]	8,08
IGF1	NM_000618	ref|Homo sapiens insulin-like growth factor 1 (somatomedin C) (IGF1), transcript variant 4, mRNA [NM_000618]	8,04
HTN3	NM_000200	ref|Homo sapiens histatin 3 (HTN3), mRNA [NM_000200]	8,03
A_32_P112263	A_32_P112263	Unknown	8,01
ROPN1B	NM_001012337	ref|Homo sapiens ropporin, rhophilin associated protein 1B (ROPN1B), mRNA [NM_001012337]	7,97
SPP1	NM_001040058	ref|Homo sapiens secreted phosphoprotein 1 (SPP1), transcript variant 1, mRNA [NM_001040058]	7,96
RP4-692D3.1	NM_001080850	ref|Homo sapiens hypothetical protein LOC728621 (LOC728621), mRNA [NM_001080850]	7,93
SPP1	NM_001040058	ref|Homo sapiens secreted phosphoprotein 1 (SPP1), transcript variant 1, mRNA [NM_001040058]	7,92
SCGB1D1	NM_006552	ref|Homo sapiens secretoglobin, family 1D, member 1 (SCGB1D1), mRNA [NM_006552]	7,89
TM4SF18	NM_138786	ref|Homo sapiens transmembrane 4 L six family member 18 (TM4SF18), mRNA [NM_138786]	7,88
SPP1	NM_001040058	ref|Homo sapiens secreted phosphoprotein 1 (SPP1), transcript variant 1, mRNA [NM_001040058]	7,85
GML	NM_002066	ref|Homo sapiens glycosylphosphatidylinositol anchored molecule like protein (GML), mRNA [NM_002066]	7,83
SPP1	NM_001040058	ref|Homo sapiens secreted phosphoprotein 1 (SPP1), transcript variant 1, mRNA [NM_001040058]	7,83
IGF1	NM_000618	ref|Homo sapiens insulin-like growth factor 1 (somatomedin C) (IGF1), transcript variant 4, mRNA [NM_000618]	7,83
ZNF585A	BC026081	gb|Homo sapiens zinc finger protein 585A, mRNA (cDNA clone IMAGE:4837344). [BC026081]	7,81
DENND2D	NM_024901	ref|Homo sapiens DENN/MADD domain containing 2D (DENND2D), mRNA [NM_024901]	7,78
BCAN	NM_021948	ref|Homo sapiens brevican (BCAN), transcript variant 1, mRNA [NM_021948]	7,71
TMPRSS3	NM_032405	ref|Homo sapiens transmembrane protease, serine 3 (TMPRSS3), transcript variant D, mRNA [NM_032405]	7,64
TSPAN10	NM_031945	ref|Homo sapiens tetraspanin 10 (TSPAN10), mRNA [NM_031945]	7,57
DNAJA4	NM_018602	ref|Homo sapiens DnaJ (Hsp40) homolog, subfamily A, member 4 (DNAJA4), transcript variant 1, mRNA [NM_018602]	7,52
IGF1	NM_000618	ref|Homo sapiens insulin-like growth factor 1 (somatomedin C) (IGF1), transcript variant 4, mRNA [NM_000618]	7,45
ELSPBP1	NM_022142	ref|Homo sapiens epididymal sperm binding protein 1 (ELSPBP1), mRNA [NM_022142]	7,45
CLDN14	NM_144492	ref|Homo sapiens claudin 14 (CLDN14), transcript variant 1, mRNA [NM_144492]	7,42
C8orf75	BC014230	gb|Homo sapiens cDNA clone IMAGE:4544942, partial cds. [BC014230]	7,27
THC2711870	THC2711870	thc|O60448_HUMAN (O60448) Neuronal thread protein AD7c-NTP, partial (5%) [THC2711870]	7,26
IGF1	NM_000618	ref|Homo sapiens insulin-like growth factor 1 (somatomedin C) (IGF1), transcript variant 4, mRNA [NM_000618]	7,25
SEMA6A	NM_020796	ref|Homo sapiens sema domain, transmembrane domain (TM), and cytoplasmic domain, (semaphorin) 6A (SEMA6A), mRNA [NM_020796]	7,14
ENST00000272035	ENST00000272035	ens|Tubulin beta-8 chain [Source:UniProtKB/Swiss-Prot;Acc:Q3ZCM7] [ENST00000272035]	7,11
RLBP1	NM_000326	ref|Homo sapiens retinaldehyde binding protein 1 (RLBP1), mRNA [NM_000326]	6,99
CRYM	NM_001888	ref|Homo sapiens crystallin, mu (CRYM), transcript variant 1, mRNA [NM_001888]	6,88
KCNAB2	NM_003636	ref|Homo sapiens potassium voltage-gated channel, shaker-related subfamily, beta member 2 (KCNAB2), transcript variant 1, mRNA [NM_003636]	6,87
CDH7	NM_004361	ref|Homo sapiens cadherin 7, type 2 (CDH7), transcript variant b, mRNA [NM_004361]	6,86
CAMK1G	NM_020439	ref|Homo sapiens calcium/calmodulin-dependent protein kinase IG (CAMK1G), mRNA [NM_020439]	6,75
RAB27A	NM_004580	ref|Homo sapiens RAB27A, member RAS oncogene family (RAB27A), transcript variant 1, mRNA [NM_004580]	6,72
CHRDL2	NM_015424	ref|Homo sapiens chordin-like 2 (CHRDL2), mRNA [NM_015424]	6,71
FGFBP2	NM_031950	ref|Homo sapiens fibroblast growth factor binding protein 2 (FGFBP2), mRNA [NM_031950]	6,70
CEACAM1	NM_001024912	ref|Homo sapiens carcinoembryonic antigen-related cell adhesion molecule 1 (biliary glycoprotein) (CEACAM1), transcript variant 2, mRNA [NM_001024912]	6,69
CA14	NM_012113	ref|Homo sapiens carbonic anhydrase XIV (CA14), mRNA [NM_012113]	6,64
D4S234E	NM_014392	ref|Homo sapiens DNA segment on chromosome 4 (unique) 234 expressed sequence (D4S234E), transcript variant 1, mRNA [NM_014392]	6,60
PPY2	NR_002181	ref|Homo sapiens pancreatic polypeptide 2 (PPY2), non-coding RNA [NR_002181]	6,57
BFSP2	NM_003571	ref|Homo sapiens beaded filament structural protein 2, phakinin (BFSP2), mRNA [NM_003571]	6,57
CAPN3	NM_000070	ref|Homo sapiens calpain 3, (p94) (CAPN3), transcript variant 1, mRNA [NM_000070]	6,56
IGF1	NM_000618	ref|Homo sapiens insulin-like growth factor 1 (somatomedin C) (IGF1), transcript variant 4, mRNA [NM_000618]	6,55
PLB1	NM_153021	ref|Homo sapiens phospholipase B1 (PLB1), mRNA [NM_153021]	6,53
SPACA3	NM_173847	ref|Homo sapiens sperm acrosome associated 3 (SPACA3), mRNA [NM_173847]	6,49

C21orf129	AK057397	gb|Homo sapiens cDNA FLJ32835 fis, clone TESTI2003255. [AK057397]	6,47
RP13-401N8.2	AL096727	gb|Homo sapiens mRNA; cDNA DKFZp434B104 (from clone DKFZp434B104). [AL096727]	6,46
ST6GALNAC2	NM_006456	ref|Homo sapiens ST6 (alpha-N-acetyl-neuraminyl-2,3-beta-galactosyl-1,3)-N-acetylgalactosaminide alpha-2,6-sialyltransferase 2 (ST6GALNAC2), mRNA [NM_(	6,42
PLP1	BC002665	gb|Homo sapiens proteolipid protein 1, mRNA (cDNA clone MGC:3940 IMAGE:3606269), complete cds. [BC002665]	6,36
FGFBP2	NM_031950	ref|Homo sapiens fibroblast growth factor binding protein 2 (FGFBP2), mRNA [NM_031950]	6,34
PRDM9	NM_020227	ref|Homo sapiens PR domain containing 9 (PRDM9), mRNA [NM_020227]	6,32
PDE4DIP	NM_014644	ref|Homo sapiens phosphodiesterase 4D interacting protein (PDE4DIP), transcript variant 1, mRNA [NM_014644]	6,29
BC014938	BC014938	gb|Homo sapiens, clone IMAGE:3922198, mRNA. [BC014938]	6,23
C1orf54	NM_024579	ref|Homo sapiens chromosome 1 open reading frame 54 (C1orf54), mRNA [NM_024579]	6,15
CARD14	NM_024110	ref|Homo sapiens caspase recruitment domain family, member 14 (CARD14), transcript variant 1, mRNA [NM_024110]	6,15
LUZP4	NM_016383	ref|Homo sapiens leucine zipper protein 4 (LUZP4), mRNA [NM_016383]	6,12
DEXI	AK055529	gb|Homo sapiens cDNA FLJ30967 fis, clone HEART2000309, weakly similar to PTB-ASSOCIATED SPLICING FACTOR. [AK055529]	6,02
DSP	NM_004415	ref|Homo sapiens desmoplakin (DSP), transcript variant 1, mRNA [NM_004415]	6,00
SCGB1D2	NM_006551	ref|Homo sapiens secretoglobin, family 1D, member 2 (SCGB1D2), mRNA [NM_006551]	5,92
ITGA7	NM_002206	ref|Homo sapiens integrin, alpha 7 (ITGA7), mRNA [NM_002206]	5,92
BIRC7	NM_022161	ref|Homo sapiens baculoviral IAP repeat-containing 7 (BIRC7), transcript variant 2, mRNA [NM_022161]	5,85
DYNC1I1	NM_004411	ref|Homo sapiens dynein, cytoplasmic 1, intermediate chain 1 (DYNC1I1), transcript variant 1, mRNA [NM_004411]	5,76
MGP	NM_000900	ref|Homo sapiens matrix Gla protein (MGP), mRNA [NM_000900]	5,67
MYLK	NM_053025	ref|Homo sapiens myosin light chain kinase (MYLK), transcript variant 1, mRNA [NM_053025]	5,67
MLLT10	BC032532	gb|Homo sapiens myeloid/lymphoid or mixed-lineage leukemia (trithorax homolog, Drosophila); translocated to, 10, mRNA (cDNA clone IMAGE:5526778), with a	5,66
THC2689164	THC2689164	Unknown	5,65
HBG1	NM_000559	ref|Homo sapiens hemoglobin, gamma A (HBG1), mRNA [NM_000559]	5,65
SEMA6A	NM_020796	ref|Homo sapiens sema domain, transmembrane domain (TM), and cytoplasmic domain, (semaphorin) 6A (SEMA6A), mRNA [NM_020796]	5,63
VGF	NM_003378	ref|Homo sapiens VGF nerve growth factor inducible (VGF), mRNA [NM_003378]	5,58
LOC645591	BC012751	gb|Homo sapiens cDNA clone IMAGE:3621376, partial cds. [BC012751]	5,56
HAS2	NM_005328	ref|Homo sapiens hyaluronan synthase 2 (HAS2), mRNA [NM_005328]	5,52
TRIM63	NM_032588	ref|Homo sapiens tripartite motif-containing 63 (TRIM63), mRNA [NM_032588]	5,52
SULT1C2	NM_176825	ref|Homo sapiens sulfotransferase family, cytosolic, 1C, member 2 (SULT1C2), transcript variant 2, mRNA [NM_176825]	5,46
LRAT	NM_004744	ref|Homo sapiens lecithin retinol acyltransferase (phosphatidylcholine--retinol O-acyltransferase) (LRAT), mRNA [NM_004744]	5,46
BHLHE41	NM_030762	ref|Homo sapiens basic helix-loop-helix domain containing, class B, 3 (BHLHB3), mRNA [NM_030762]	5,40
THC2733597	THC2733597	thc|Q40J89_EHRCH (Q40J89) Cation efflux protein, partial (6%) [THC2733597]	5,33
THC2701341	THC2701341	thc|DQ071571 spermatin {Rattus norvegicus} (exp=-1; wgp=0; cg=0), partial (19%) [THC2701341]	5,28
SEMA6A	NM_020796	ref|Homo sapiens sema domain, transmembrane domain (TM), and cytoplasmic domain, (semaphorin) 6A (SEMA6A), mRNA [NM_020796]	5,27
SGCD	NM_000337	ref|Homo sapiens sarcoglycan, delta (35kDa dystrophin-associated glycoprotein) (SGCD), transcript variant 1, mRNA [NM_000337]	5,25
hCG_2019139	AK056971	gb|Homo sapiens cDNA FLJ32409 fis, clone SKMUS2000468. [AK056971]	5,23
AF339787	AF339787	gb|Homo sapiens clone IMAGE:205688, mRNA sequence. [AF339787]	5,18
CBX5	NM_001127322	ref|Homo sapiens chromobox homolog 5 (HP1 alpha homolog, Drosophila) (CBX5), transcript variant 1, mRNA [NM_001127322]	5,18
ST3GAL4	NM_006278	ref|Homo sapiens ST3 beta-galactoside alpha-2,3-sialyltransferase 4 (ST3GAL4), mRNA [NM_006278]	5,17
BCAN	NM_198427	ref|Homo sapiens brevican (BCAN), transcript variant 2, mRNA [NM_198427]	5,13
CTSZ	NM_001336	ref|Homo sapiens cathepsin Z (CTSZ), mRNA [NM_001336]	5,12
MITF	NM_198159	ref|Homo sapiens microphthalmia-associated transcription factor (MITF), transcript variant 1, mRNA [NM_198159]	5,11
ATOH8	NM_032827	ref|Homo sapiens atonal homolog 8 (Drosophila) (ATOH8), mRNA [NM_032827]	5,08
MITF	NM_198159	ref|Homo sapiens microphthalmia-associated transcription factor (MITF), transcript variant 1, mRNA [NM_198159]	5,07
GALNT3	NM_004482	ref|Homo sapiens UDP-N-acetyl-alpha-D-galactosamine:polypeptide N-acetylgalactosaminyltransferase 3 (GalNAc-T3) (GALNT3), mRNA [NM_004482]	5,01
CDK2	NM_001798	ref|Homo sapiens cyclin-dependent kinase 2 (CDK2), transcript variant 1, mRNA [NM_001798]	4,98
TYRP1	NM_000550	ref|Homo sapiens tyrosinase-related protein 1 (TYRP1), mRNA [NM_000550]	4,98
LOC286382	AK094356	gb|Homo sapiens cDNA FLJ37037 fis, clone BRACE2011611. [AK094356]	4,97
DYNC1I1	NM_004411	ref|Homo sapiens dynein, cytoplasmic 1, intermediate chain 1 (DYNC1I1), transcript variant 1, mRNA [NM_004411]	4,96
PMP2	NM_002677	ref|Homo sapiens peripheral myelin protein 2 (PMP2), mRNA [NM_002677]	4,89
BACE2	NM_012105	ref|Homo sapiens beta-site APP-cleaving enzyme 2 (BACE2), transcript variant a, mRNA [NM_012105]	4,87
SOX10	NM_006941	ref|Homo sapiens SRY (sex determining region Y)-box 10 (SOX10), mRNA [NM_006941]	4,85
SOX10	NM_006941	ref|Homo sapiens SRY (sex determining region Y)-box 10 (SOX10), mRNA [NM_006941]	4,85
CDK2	NM_001798	ref|Homo sapiens cyclin-dependent kinase 2 (CDK2), transcript variant 1, mRNA [NM_001798]	4,83
CDK2	NM_001798	ref|Homo sapiens cyclin-dependent kinase 2 (CDK2), transcript variant 1, mRNA [NM_001798]	4,82
MYLK	NM_053025	ref|Homo sapiens myosin light chain kinase (MYLK), transcript variant 1, mRNA [NM_053025]	4,82
PHF15	NM_015288	ref|Homo sapiens PHD finger protein 15 (PHF15), mRNA [NM_015288]	4,81
GPR161	NM_153832	ref|Homo sapiens G protein-coupled receptor 161 (GPR161), transcript variant 2, mRNA [NM_153832]	4,81
PHF15	NM_015288	ref|Homo sapiens PHD finger protein 15 (PHF15), mRNA [NM_015288]	4,80
C16orf89	NM_152459	ref|Homo sapiens chromosome 16 open reading frame 89 (C16orf89), transcript variant 1, mRNA [NM_152459]	4,80
SIRPB1	NM_006065	ref|Homo sapiens signal-regulatory protein beta 1 (SIRPB1), transcript variant 1, mRNA [NM_006065]	4,79
BACE2	NM_012105	ref|Homo sapiens beta-site APP-cleaving enzyme 2 (BACE2), transcript variant a, mRNA [NM_012105]	4,78
SIRPA	NM_001040022	ref|Homo sapiens signal-regulatory protein alpha (SIRPA), transcript variant 1, mRNA [NM_001040022]	4,78
KCNAB1	NM_003471	ref|Homo sapiens potassium voltage-gated channel, shaker-related subfamily, beta member 1 (KCNAB1), transcript variant 2, mRNA [NM_003471]	4,78
CDK2	NM_001798	ref|Homo sapiens cyclin-dependent kinase 2 (CDK2), transcript variant 1, mRNA [NM_001798]	4,76
PKD1L2	NM_052892	ref|Homo sapiens polycystic kidney disease 1-like 2 (PKD1L2), transcript variant 1, mRNA [NM_052892]	4,75
HBG1	NM_000559	ref|Homo sapiens hemoglobin, gamma A (HBG1), mRNA [NM_000559]	4,75
CDK2	NM_001798	ref|Homo sapiens cyclin-dependent kinase 2 (CDK2), transcript variant 1, mRNA [NM_001798]	4,75
KCNAB2	NM_003636	ref|Homo sapiens potassium voltage-gated channel, shaker-related subfamily, beta member 2 (KCNAB2), transcript variant 1, mRNA [NM_003636]	4,72
PPP1CB	NM_002709	ref|Homo sapiens protein phosphatase 1, catalytic subunit, beta isoform (PPP1CB), transcript variant 1, mRNA [NM_002709]	4,72
C6orf218	NM_152738	ref|Homo sapiens chromosome 6 open reading frame 218 (C6orf218), mRNA [NM_152738]	4,70
ENST00000327299	ENST00000327299	ens|Adenylate kinase isoenzyme 4, mitochondrial (EC 2.7.4.3)(Adenylate kinase 3-like)(ATP-AMP transphosphorylase) [Source:UniProtKB/Swiss-Prot;Acc:P271·	4,70
GHR	NM_000163	ref|Homo sapiens growth hormone receptor (GHR), mRNA [NM_000163]	4,68
PPARGC1A	NM_013261	ref|Homo sapiens peroxisome proliferator-activated receptor gamma, coactivator 1 alpha (PPARGC1A), mRNA [NM_013261]	4,67
HBG1	NM_000559	ref|Homo sapiens hemoglobin, gamma A (HBG1), mRNA [NM_000559]	4,67
SLIT1	NM_003061	ref|Homo sapiens slit homolog 1 (Drosophila) (SLIT1), mRNA [NM_003061]	4,66
PTCRA	NM_138296	ref|Homo sapiens pre T-cell antigen receptor alpha (PTCRA), mRNA [NM_138296]	4,64
MOBKL2B	NM_024761	ref|Homo sapiens MOB1, Mps One Binder kinase activator-like 2B (yeast) (MOBKL2B), mRNA [NM_024761]	4,62
CDK2	NM_001798	ref|Homo sapiens cyclin-dependent kinase 2 (CDK2), transcript variant 1, mRNA [NM_001798]	4,61
MLPH	NM_024101	ref|Homo sapiens melanophilin (MLPH), transcript variant 1, mRNA [NM_024101]	4,61
GJB1	NM_000166	ref|Homo sapiens gap junction protein, beta 1, 32kDa (GJB1), transcript variant 2, mRNA [NM_000166]	4,60
MCAM	NM_006500	ref|Homo sapiens melanoma cell adhesion molecule (MCAM), mRNA [NM_006500]	4,59
CDK2	NM_001798	ref|Homo sapiens cyclin-dependent kinase 2 (CDK2), transcript variant 1, mRNA [NM_001798]	4,59
LEPREL1	NM_018192	ref|Homo sapiens leprecan-like 1 (LEPREL1), transcript variant 1, mRNA [NM_018192]	4,59
MITF	NM_198159	ref|Homo sapiens microphthalmia-associated transcription factor (MITF), transcript variant 1, mRNA [NM_198159]	4,58
ENO2	NM_001975	ref|Homo sapiens enolase 2 (gamma, neuronal) (ENO2), mRNA [NM_001975]	4,58
DHRS2	NM_182908	ref|Homo sapiens dehydrogenase/reductase (SDR family) member 2 (DHRS2), transcript variant 1, mRNA [NM_182908]	4,58
HBG1	NM_000559	ref|Homo sapiens hemoglobin, gamma A (HBG1), mRNA [NM_000559]	4,57
CDK2	NM_001798	ref|Homo sapiens cyclin-dependent kinase 2 (CDK2), transcript variant 1, mRNA [NM_001798]	4,55
CV800149	CV800149	gb|AGENCOURT_37001201 NIH_MGC_280 Homo sapiens cDNA clone IMAGE:7505458 5', mRNA sequence [CV800149]	4,49
SPRR3	NM_005416	ref|Homo sapiens small proline-rich protein 3 (SPRR3), transcript variant 1, mRNA [NM_005416]	4,48
ITK	NM_005546	ref|Homo sapiens IL2-inducible T-cell kinase (ITK), mRNA [NM_005546]	4,48
KIAA0746	NM_015187	ref|Homo sapiens KIAA0746 protein (KIAA0746), mRNA [NM_015187]	4,48
THC2520542	THC2520542	thc|Q9XDH2_MYCTU (Q9XDH2) Proline-rich mucin homolog, partial (3%) [THC2520542]	4,48
ATP10A	NM_024490	ref|Homo sapiens ATPase, class V, type 10A (ATP10A), mRNA [NM_024490]	4,46
ST3GAL4	AK021929	gb|Homo sapiens cDNA FLJ11867 fis, clone HEMBA1006976, weakly similar to H.sapiens mRNA for Gal-beta(1-3/1-4)GlcNAc alpha-2.3-sialyltransferase. [AK02	4,45
HBG1	NM_000559	ref|Homo sapiens hemoglobin, gamma A (HBG1), mRNA [NM_000559]	4,44
GJB1	NM_000166	ref|Homo sapiens gap junction protein, beta 1, 32kDa (GJB1), transcript variant 2, mRNA [NM_000166]	4,44
MYH10	NM_005964	ref|Homo sapiens myosin, heavy chain 10, non-muscle (MYH10), mRNA [NM_005964]	4,43
DUSP23	NM_017823	ref|Homo sapiens dual specificity phosphatase 23 (DUSP23), mRNA [NM_017823]	4,43
CDK2	NM_001798	ref|Homo sapiens cyclin-dependent kinase 2 (CDK2), transcript variant 1, mRNA [NM_001798]	4,43
PKIA	NM_006823	ref|Homo sapiens protein kinase (cAMP-dependent, catalytic) inhibitor alpha (PKIA), transcript variant 6, mRNA [NM_006823]	4,40
RGN	NM_152869	ref|Homo sapiens regucalcin (senescence marker protein-30) (RGN), transcript variant 2, mRNA [NM_152869]	4,40
CCL17	NM_002987	ref|Homo sapiens chemokine (C-C motif) ligand 17 (CCL17), mRNA [NM_002987]	4,38
OPHN1	NM_002547	ref|Homo sapiens oligophrenin 1 (OPHN1), mRNA [NM_002547]	4,38
FAM174B	NM_207446	ref|Homo sapiens family with sequence similarity 174, member B (FAM174B), mRNA [NM_207446]	4,37
FSTL5	NM_020116	ref|Homo sapiens follistatin-like 5 (FSTL5), transcript variant 1, mRNA [NM_020116]	4,34
MCAM	NM_006500	ref|Homo sapiens melanoma cell adhesion molecule (MCAM), mRNA [NM_006500]	4,34
HBG1	NM_000559	ref|Homo sapiens hemoglobin, gamma A (HBG1), mRNA [NM_000559]	4,32
DHRS2	NM_182908	ref|Homo sapiens dehydrogenase/reductase (SDR family) member 2 (DHRS2), transcript variant 1, mRNA [NM_182908]	4,32
ST6GALNAC1	NM_018414	ref|Homo sapiens ST6 (alpha-N-acetyl-neuraminyl-2,3-beta-galactosyl-1,3)-N-acetylgalactosaminide alpha-2,6-sialyltransferase 1 (ST6GALNAC1), mRNA [NM_(	4,29
NAT8B	NM_016347	ref|Homo sapiens N-acetyltransferase 8B (GCN5-related, putative, gene/pseudogene) (NAT8B), mRNA [NM_016347]	4,26

PMP2	NM_002677	ref|Homo sapiens peripheral myelin protein 2 (PMP2), mRNA [NM_002677]	4,26
HBG1	NM_000559	ref|Homo sapiens hemoglobin, gamma A (HBG1), mRNA [NM_000559]	4,25
GAGE3	U19144	gb|Human GAGE-3 protein mRNA, complete cds. [U19144]	4,25
LDB3	NM_007078	ref|Homo sapiens LIM domain binding 3 (LDB3), transcript variant 1, mRNA [NM_007078]	4,24
CDK2	NM_001798	ref|Homo sapiens cyclin-dependent kinase 2 (CDK2), transcript variant 1, mRNA [NM_001798]	4,24
FCRLA	NM_032738	ref|Homo sapiens Fc receptor-like A (FCRLA), mRNA [NM_032738]	4,24
BST2	NM_004335	ref|Homo sapiens bone marrow stromal cell antigen 2 (BST2), mRNA [NM_004335]	4,22
AP1S2	NM_003916	ref|Homo sapiens adaptor-related protein complex 1, sigma 2 subunit (AP1S2), mRNA [NM_003916]	4,22
SILV	NM_006928	ref|Homo sapiens silver homolog (mouse) (SILV), mRNA [NM_006928]	4,21
SIRPA	NM_001040022	ref|Homo sapiens signal-regulatory protein alpha (SIRPA), transcript variant 1, mRNA [NM_001040022]	4,19
MLPH	NM_001042467	ref|Homo sapiens melanophilin (MLPH), transcript variant 2, mRNA [NM_001042467]	4,19
CR749704	CR749704	gb|Homo sapiens mRNA; cDNA DKFZp434D1072 (from clone DKFZp434D1072). [CR749704]	4,17
BC063881	BC063881	gb|Homo sapiens cDNA clone IMAGE:30390303, with apparent retained intron. [BC063881]	4,14
SLC45A2	NM_016180	ref|Homo sapiens solute carrier family 45, member 2 (SLC45A2), transcript variant 1, mRNA [NM_016180]	4,12
HBG1	NM_000559	ref|Homo sapiens hemoglobin, gamma A (HBG1), mRNA [NM_000559]	4,11
DMKN	NM_001035516	ref|Homo sapiens dermokine (DMKN), transcript variant 1, mRNA [NM_001035516]	4,10
STK32A	NM_145001	ref|Homo sapiens serine/threonine kinase 32A (STK32A), transcript variant 2, mRNA [NM_145001]	4,09
FAM167B	NM_032648	ref|Homo sapiens family with sequence similarity 167, member B (FAM167B), mRNA [NM_032648]	4,08
AK024680	AK024680	gb|Homo sapiens cDNA: FLJ21027 fis, clone CAE07110. [AK024680]	4,05
MOBKL2B	NM_024761	ref|Homo sapiens MOB1, Mps One Binder kinase activator-like 2B (yeast) (MOBKL2B), mRNA [NM_024761]	4,04
UROS	CR601670	gb|full-length cDNA clone CS0DL001YD09 of B cells (Ramos cell line) Cot 25-normalized of Homo sapiens (human). [CR601670]	4,04
VEGFB	NM_003377	ref|Homo sapiens vascular endothelial growth factor B (VEGFB), mRNA [NM_003377]	4,04
TTC39A	NM_001080494	ref|Homo sapiens tetratricopeptide repeat domain 39A (TTC39A), mRNA [NM_001080494]	4,03
HBG1	NM_000559	ref|Homo sapiens hemoglobin, gamma A (HBG1), mRNA [NM_000559]	4,02
CEACAM3	NM_001815	ref|Homo sapiens carcinoembryonic antigen-related cell adhesion molecule 3 (CEACAM3), mRNA [NM_001815]	4,01
HBG1	NM_000559	ref|Homo sapiens hemoglobin, gamma A (HBG1), mRNA [NM_000559]	4,00
GPR161	NM_153832	ref|Homo sapiens G protein-coupled receptor 161 (GPR161), transcript variant 2, mRNA [NM_153832]	4,00
ITM2A	NM_004867	ref|Homo sapiens integral membrane protein 2A (ITM2A), mRNA [NM_004867]	4,00
LRAT	NM_004744	ref|Homo sapiens lecithin retinol acyltransferase (phosphatidylcholine--retinol O-acyltransferase) (LRAT), mRNA [NM_004744]	3,99
MLPH	NM_024101	ref|Homo sapiens melanophilin (MLPH), transcript variant 1, mRNA [NM_024101]	3,98
FUT5	NM_002034	ref|Homo sapiens fucosyltransferase 5 (alpha (1,3) fucosyltransferase) (FUT5), mRNA [NM_002034]	3,97
GPR56	NM_201525	ref|Homo sapiens G protein-coupled receptor 56 (GPR56), transcript variant 3, mRNA [NM_201525]	3,96
PPM1H	NM_020700	ref|Homo sapiens protein phosphatase 1H (PP2C domain containing) (PPM1H), mRNA [NM_020700]	3,95
HBG1	NM_000559	ref|Homo sapiens hemoglobin, gamma A (HBG1), mRNA [NM_000559]	3,94
ME3	NM_001014811	ref|Homo sapiens malic enzyme 3, NADP(+)-dependent, mitochondrial (ME3), nuclear gene encoding mitochondrial protein, transcript variant 2, mRNA [NM_001	3,93
GPR161	NM_153832	ref|Homo sapiens G protein-coupled receptor 161 (GPR161), transcript variant 2, mRNA [NM_153832]	3,92
E2F1	NM_005225	ref|Homo sapiens E2F transcription factor 1 (E2F1), mRNA [NM_005225]	3,92
ENST00000327299	ENST00000327299	ens|Adenylate kinase isoenzyme 4, mitochondrial (EC 2.7.4.3)(Adenylate kinase 3-like)(ATP-AMP transphosphorylase) [Source:UniProtKB/Swiss-Prot;Acc:P271	3,91
AX721193	AX721193	gb|Sequence 153 from Patent WO0220754. [AX721193]	3,91
MDGA1	NM_153487	ref|Homo sapiens MAM domain containing glycosylphosphatidylinositol anchor 1 (MDGA1), mRNA [NM_153487]	3,91
SMPD1	NM_000543	ref|Homo sapiens sphingomyelin phosphodiesterase 1, acid lysosomal (SMPD1), transcript variant 1, mRNA [NM_000543]	3,89
E2F1	NM_005225	ref|Homo sapiens E2F transcription factor 1 (E2F1), mRNA [NM_005225]	3,89
ANXA13	NM_001003954	ref|Homo sapiens annexin A13 (ANXA13), transcript variant 2, mRNA [NM_001003954]	3,88
BI013473	BI013473	gb|RC0-ET0185-220101-021-h02 ET0185 Homo sapiens cDNA, mRNA sequence [BI013473]	3,88
E2F1	NM_005225	ref|Homo sapiens E2F transcription factor 1 (E2F1), mRNA [NM_005225]	3,87
BC061638	BC061638	gb|Homo sapiens cDNA clone IMAGE:5547707, partial cds. [BC061638]	3,86
E2F1	NM_005225	ref|Homo sapiens E2F transcription factor 1 (E2F1), mRNA [NM_005225]	3,86
STXBP6	NM_014178	ref|Homo sapiens syntaxin binding protein 6 (amisyn) (STXBP6), mRNA [NM_014178]	3,85
LRGUK	NM_144648	ref|Homo sapiens leucine-rich repeats and guanylate kinase domain containing (LRGUK), mRNA [NM_144648]	3,85
GAGE7	NM_021123	ref|Homo sapiens G antigen 7 (GAGE7), mRNA [NM_021123]	3,84
MORC1	NM_014429	ref|Homo sapiens MORC family CW-type zinc finger 1 (MORC1), mRNA [NM_014429]	3,84
PTPRJ	NM_002843	ref|Homo sapiens protein tyrosine phosphatase, receptor type, J (PTPRJ), transcript variant 1, mRNA [NM_002843]	3,84
E2F1	NM_005225	ref|Homo sapiens E2F transcription factor 1 (E2F1), mRNA [NM_005225]	3,83
FYB	NM_001465	ref|Homo sapiens FYN binding protein (FYB-120/130) (FYB), transcript variant 1, mRNA [NM_001465]	3,83
AK090694	AK090694	gb|Homo sapiens cDNA FLJ33375 fis, clone BRACE2006137. [AK090694]	3,83
E2F1	NM_005225	ref|Homo sapiens E2F transcription factor 1 (E2F1), mRNA [NM_005225]	3,83
CBLN1	NM_004352	ref|Homo sapiens cerebellin 1 precursor (CBLN1), mRNA [NM_004352]	3,82
GHR	NM_000163	ref|Homo sapiens growth hormone receptor (GHR), mRNA [NM_000163]	3,82
SCARB1	NM_005505	ref|Homo sapiens scavenger receptor class B, member 1 (SCARB1), transcript variant 1, mRNA [NM_005505]	3,81
PRDM9	NM_020227	ref|Homo sapiens PR domain containing 9 (PRDM9), mRNA [NM_020227]	3,79
HLA-DMB	NM_002118	ref|Homo sapiens major histocompatibility complex, class II, DM beta (HLA-DMB), mRNA [NM_002118]	3,79
DHRS3	NM_004753	ref|Homo sapiens dehydrogenase/reductase (SDR family) member 3 (DHRS3), mRNA [NM_004753]	3,77
PKD1L2	NM_052892	ref|Homo sapiens polycystic kidney disease 1-like 2 (PKD1L2), transcript variant 1, mRNA [NM_052892]	3,77
SNAI2	NM_003068	ref|Homo sapiens snail homolog 2 (Drosophila) (SNAI2), mRNA [NM_003068]	3,76
E2F1	NM_005225	ref|Homo sapiens E2F transcription factor 1 (E2F1), mRNA [NM_005225]	3,76
E2F1	NM_005225	ref|Homo sapiens E2F transcription factor 1 (E2F1), mRNA [NM_005225]	3,76
ITGB8	NM_002214	ref|Homo sapiens integrin, beta 8 (ITGB8), mRNA [NM_002214]	3,75
EDEM1	NM_014674	ref|Homo sapiens ER degradation enhancer, mannosidase alpha-like 1 (EDEM1), mRNA [NM_014674]	3,75
RGS20	NM_170587	ref|Homo sapiens regulator of G-protein signaling 20 (RGS20), transcript variant 1, mRNA [NM_170587]	3,73
CABLES1	NM_138375	ref|Homo sapiens Cdk5 and Abl enzyme substrate 1 (CABLES1), transcript variant 1, mRNA [NM_138375]	3,73
IRF4	NM_002460	ref|Homo sapiens interferon regulatory factor 4 (IRF4), mRNA [NM_002460]	3,72
E2F1	NM_005225	ref|Homo sapiens E2F transcription factor 1 (E2F1), mRNA [NM_005225]	3,71
E2F1	NM_005225	ref|Homo sapiens E2F transcription factor 1 (E2F1), mRNA [NM_005225]	3,71
EPHX1	NM_000120	ref|Homo sapiens epoxide hydrolase 1, microsomal (xenobiotic) (EPHX1), transcript variant 1, mRNA [NM_000120]	3,71
GPR56	NM_201525	ref|Homo sapiens G protein-coupled receptor 56 (GPR56), transcript variant 3, mRNA [NM_201525]	3,69
LYPD1	NM_144586	ref|Homo sapiens LY6/PLAUR domain containing 1 (LYPD1), transcript variant 1, mRNA [NM_144586]	3,69
FAM152A	BC020640	gb|Homo sapiens family with sequence similarity 152, member A, mRNA (cDNA clone IMAGE:4718788), partial cds. [BC020640]	3,69
KRT23	NM_015515	ref|Homo sapiens keratin 23 (histone deacetylase inducible) (KRT23), mRNA [NM_015515]	3,68
PTTG1IP	NM_004339	ref|Homo sapiens pituitary tumor-transforming 1 interacting protein (PTTG1IP), mRNA [NM_004339]	3,68
X83127	X83127	gb|H.sapiens mRNA for voltage gated potassium channels, beta subunit. [X83127]	3,68
PLXNC1	AB208934	gb|Homo sapiens mRNA for plexin C1 variant protein. [AB208934]	3,68
TMPRSS5	NM_030770	ref|Homo sapiens transmembrane protease, serine 5 (TMPRSS5), mRNA [NM_030770]	3,68
SMOC2	NM_022138	ref|Homo sapiens SPARC related modular calcium binding 2 (SMOC2), mRNA [NM_022138]	3,67
NRP2	NM_201266	ref|Homo sapiens neuropilin 2 (NRP2), transcript variant 1, mRNA [NM_201266]	3,67
BG682198	BG682198	gb|602629505F1 NCI_CGAP_Skn4 Homo sapiens cDNA clone IMAGE:4754377 5', mRNA sequence [BG682198]	3,67
SFTPC	NM_003018	ref|Homo sapiens surfactant protein C (SFTPC), mRNA [NM_003018]	3,67
C15orf39	NM_015492	ref|Homo sapiens chromosome 15 open reading frame 39 (C15orf39), mRNA [NM_015492]	3,66
AFAP1L1	NM_152406	ref|Homo sapiens actin filament associated protein 1-like 1 (AFAP1L1), mRNA [NM_152406]	3,66
CDH7	NM_004361	ref|Homo sapiens cadherin 7, type 2 (CDH7), transcript variant b, mRNA [NM_004361]	3,65
DENND2A	NM_015689	ref|Homo sapiens DENN/MADD domain containing 2A (DENND2A), mRNA [NM_015689]	3,65
S1PR4	NM_003775	ref|Homo sapiens sphingosine-1-phosphate receptor 4 (S1PR4), mRNA [NM_003775]	3,65
OR7A5	NM_017506	ref|Homo sapiens olfactory receptor, family 7, subfamily A, member 5 (OR7A5), mRNA [NM_017506]	3,64
KEL	NM_000420	ref|Homo sapiens Kell blood group, metallo-endopeptidase (KEL), mRNA [NM_000420]	3,64
C9orf93	NM_173550	ref|Homo sapiens chromosome 9 open reading frame 93 (C9orf93), mRNA [NM_173550]	3,63
LOC390595	AK023802	gb|Homo sapiens cDNA FLJ13740 fis, clone PLACE3000199. [AK023802]	3,61
THC2689504	THC2689504	thc|Q5E4L1_VIBF1 (Q5E4L1) Suppressor/enhancer of lin-12, partial (6%) [THC2689504]	3,61
AA778291	AA778291	gb|AA778291 zl56d07.s1 Soares_pregnant_uterus_NbHPU Homo sapiens cDNA clone IMAGE:505933 3', mRNA sequence [AA778291]	3,60
WHSC1	NM_007331	ref|Homo sapiens Wolf-Hirschhorn syndrome candidate 1 (WHSC1), transcript variant 8, mRNA [NM_007331]	3,60
OLFM1	NM_014279	ref|Homo sapiens olfactomedin 1 (OLFM1), transcript variant 1, mRNA [NM_014279]	3,59
LGI3	NM_139278	ref|Homo sapiens leucine-rich repeat LGI family, member 3 (LGI3), mRNA [NM_139278]	3,58
CARD14	NM_052819	ref|Homo sapiens caspase recruitment domain family, member 14 (CARD14), transcript variant 2, mRNA [NM_052819]	3,57
AP1S2	NM_003916	ref|Homo sapiens adaptor-related protein complex 1, sigma 2 subunit (AP1S2), mRNA [NM_003916]	3,56
SSU72	AK023110	gb|Homo sapiens cDNA FLJ13048 fis, clone NT2RP3001399, weakly similar to SSU72 PROTEIN. [AK023110]	3,54
SCARB1	NM_005505	ref|Homo sapiens scavenger receptor class B, member 1 (SCARB1), transcript variant 1, mRNA [NM_005505]	3,53
NAV2	NM_182964	ref|Homo sapiens neuron navigator 2 (NAV2), transcript variant 1, mRNA [NM_182964]	3,51
PPARGC1A	NM_013261	ref|Homo sapiens peroxisome proliferator-activated receptor gamma, coactivator 1 alpha (PPARGC1A), mRNA [NM_013261]	3,51
CNIH3	NM_152495	ref|Homo sapiens cornichon homolog 3 (Drosophila) (CNIH3), mRNA [NM_152495]	3,50
TYR	NM_000372	ref|Homo sapiens tyrosinase (oculocutaneous albinism IA) (TYR), mRNA [NM_000372]	3,49
FSTL4	NM_015082	ref|Homo sapiens follistatin-like 4 (FSTL4), mRNA [NM_015082]	3,48
NCALD	NM_001040630	ref|Homo sapiens neurocalcin delta (NCALD), transcript variant 7, mRNA [NM_001040630]	3,47
VEGFB	NM_003377	ref|Homo sapiens vascular endothelial growth factor B (VEGFB), mRNA [NM_003377]	3,44

AK021785	AK021785	gb|Homo sapiens cDNA FLJ11723 fis, clone HEMBA1005314. [AK021785]	3,44
CPB1	NM_001871	ref|Homo sapiens carboxypeptidase B1 (tissue) (CPB1), mRNA [NM_001871]	3,43
HSPB2	NM_001541	ref|Homo sapiens heat shock 27kDa protein 2 (HSPB2), mRNA [NM_001541]	3,42
NR4A3	NM_173198	ref|Homo sapiens nuclear receptor subfamily 4, group A, member 3 (NR4A3), transcript variant 2, mRNA [NM_173198]	3,41
GAS7	NM_201433	ref|Homo sapiens growth arrest-specific 7 (GAS7), transcript variant c, mRNA [NM_201433]	3,41
AF086187	AF086187	gb|Homo sapiens full length insert cDNA clone ZC30H06. [AF086187]	3,41
IGSF8	NM_052868	ref|Homo sapiens immunoglobulin superfamily, member 8 (IGSF8), mRNA [NM_052868]	3,41
DHRS13	NM_144683	ref|Homo sapiens dehydrogenase/reductase (SDR family) member 13 (DHRS13), mRNA [NM_144683]	3,39
SEMA7A	NM_003612	ref|Homo sapiens semaphorin 7A, GPI membrane anchor (John Milton Hagen blood group) (SEMA7A), mRNA [NM_003612]	3,38
MBOAT1	NM_001080480	ref|Homo sapiens membrane bound O-acyltransferase domain containing 1 (MBOAT1), mRNA [NM_001080480]	3,38
SILV	NM_006928	ref|Homo sapiens silver homolog (mouse) (SILV), mRNA [NM_006928]	3,37
ATP8A2	NM_016529	ref|Homo sapiens ATPase, aminophospholipid transporter-like, class I, type 8A, member 2 (ATP8A2), mRNA [NM_016529]	3,37
KIAA1598	NM_018330	ref|Homo sapiens KIAA1598 (KIAA1598), transcript variant 2, mRNA [NM_018330]	3,37
OSBPL10	NM_017784	ref|Homo sapiens oxysterol binding protein-like 10 (OSBPL10), mRNA [NM_017784]	3,37
SLAMF8	NM_020125	ref|Homo sapiens SLAM family member 8 (SLAMF8), mRNA [NM_020125]	3,36
ECM1	NM_004425	ref|Homo sapiens extracellular matrix protein 1 (ECM1), transcript variant 1, mRNA [NM_004425]	3,35
CFH	NM_001014975	ref|Homo sapiens complement factor H (CFH), transcript variant 2, mRNA [NM_001014975]	3,35
NAP5	NM_207363	ref|Homo sapiens Nck-associated protein 5 (NAP5), transcript variant 1, mRNA [NM_207363]	3,35
CA12	NM_001218	ref|Homo sapiens carbonic anhydrase XII (CA12), transcript variant 1, mRNA [NM_001218]	3,34
FLJ21075	NM_025031	ref|Homo sapiens hypothetical protein FLJ21075 (FLJ21075), mRNA [NM_025031]	3,33
C21orf90	NR_026547	ref|Homo sapiens chromosome 21 open reading frame 90 (C21orf90), transcript variant 1, non-coding RNA [NR_026547]	3,33
TYR	NM_000372	ref|Homo sapiens tyrosinase (oculocutaneous albinism IA) (TYR), mRNA [NM_000372]	3,33
RASSF4	NM_032023	ref|Homo sapiens Ras association (RalGDS/AF-6) domain family member 4 (RASSF4), mRNA [NM_032023]	3,32
FBXO2	NM_012168	ref|Homo sapiens F-box protein 2 (FBXO2), mRNA [NM_012168]	3,31
GDPD5	NM_030792	ref|Homo sapiens glycerophosphodiester phosphodiesterase domain containing 5 (GDPD5), mRNA [NM_030792]	3,31
JRK	NM_003724	ref|Homo sapiens jerky homolog (mouse) (JRK), transcript variant 1, mRNA [NM_003724]	3,31
ENST00000327299	ENST00000327299	ens|Adenylate kinase isoenzyme 4, mitochondrial (EC 2.7.4.3)(Adenylate kinase 3-like)(ATP-AMP transphosphorylase) [Source:UniProtKB/Swiss-Prot;Acc:P271	3,29
CITED1	NM_004143	ref|Homo sapiens Cbp/p300-interacting transactivator, with Glu/Asp-rich carboxy-terminal domain, 1 (CITED1), mRNA [NM_004143]	3,29
GDPD5	NM_030792	ref|Homo sapiens glycerophosphodiester phosphodiesterase domain containing 5 (GDPD5), mRNA [NM_030792]	3,28
FIGNL1	NM_001042762	ref|Homo sapiens fidgetin-like 1 (FIGNL1), transcript variant 1, mRNA [NM_001042762]	3,28
DYNLT3	NM_006520	ref|Homo sapiens dynein, light chain, Tctex-type 3 (DYNLT3), mRNA [NM_006520]	3,28
CKB	NM_001823	ref|Homo sapiens creatine kinase, brain (CKB), mRNA [NM_001823]	3,28
AMDHD2	NM_015944	ref|Homo sapiens amidohydrolase domain containing 2 (AMDHD2), mRNA [NM_015944]	3,27
TRPM3	NM_001007471	ref|Homo sapiens transient receptor potential cation channel, subfamily M, member 3 (TRPM3), transcript variant 9, mRNA [NM_001007471]	3,27
GPR143	NM_000273	ref|Homo sapiens G protein-coupled receptor 143 (GPR143), mRNA [NM_000273]	3,27
THC2539492	THC2539492	thc|CT091_HUMAN (Q5T1J6) Protein C20orf91, partial (66%) [THC2539492]	3,26
MAGEB6	NM_173523	ref|Homo sapiens melanoma antigen family B, 6 (MAGEB6), mRNA [NM_173523]	3,25
ENPP1	NM_006208	ref|Homo sapiens ectonucleotide pyrophosphatase/phosphodiesterase 1 (ENPP1), mRNA [NM_006208]	3,25
PMP2	NM_002677	ref|Homo sapiens peripheral myelin protein 2 (PMP2), mRNA [NM_002677]	3,25
ATP6V0D2	NM_152565	ref|Homo sapiens ATPase, H+ transporting, lysosomal 38kDa, V0 subunit d2 (ATP6V0D2), mRNA [NM_152565]	3,24
CFH	NM_000186	ref|Homo sapiens complement factor H (CFH), transcript variant 1, mRNA [NM_000186]	3,24
C1orf133	NR_024337	ref|Homo sapiens chromosome 1 open reading frame 133 (C1orf133), non-coding RNA [NR_024337]	3,24
DENND2A	NM_015689	ref|Homo sapiens DENN/MADD domain containing 2A (DENND2A), mRNA [NM_015689]	3,23
MLLT10	NM_001009569	ref|Homo sapiens myeloid/lymphoid or mixed-lineage leukemia (trithorax homolog, Drosophila); translocated to, 10 (MLLT10), transcript variant 2, mRNA [NM_00	3,23
ENST00000327506	ENST00000327506	ens|Protein LOC339766 [Source:UniProtKB/Swiss-Prot;Acc:A6NES4] [ENST00000327506]	3,22
BC047326	BC047326	gb|Homo sapiens, clone IMAGE:5528960, mRNA. [BC047326]	3,21
MAP7D2	NM_152780	ref|Homo sapiens MAP7 domain containing 2 (MAP7D2), mRNA [NM_152780]	3,20
NCRNA00084	U60873	gb|Human clone 137308 mRNA, partial cds. [U60873]	3,20
GYPC	NM_002101	ref|Homo sapiens glycophorin C (Gerbich blood group) (GYPC), transcript variant 1, mRNA [NM_002101]	3,19
FAM75A2	NM_001040065	ref|Homo sapiens family with sequence similarity 75, member A2 (FAM75A2), mRNA [NM_001040065]	3,19
STAC	NM_003149	ref|Homo sapiens SH3 and cysteine rich domain (STAC), mRNA [NM_003149]	3,18
CR620043	CR620043	gb|full-length cDNA clone CS0DC001YP21 of Neuroblastoma Cot 25-normalized of Homo sapiens (human). [CR620043]	3,18
SCML4	NM_198081	ref|Homo sapiens sex comb on midleg-like 4 (Drosophila) (SCML4), mRNA [NM_198081]	3,18
TNS3	NM_022748	ref|Homo sapiens tensin 3 (TNS3), mRNA [NM_022748]	3,18
ANKRD18A	BC131497	gb|Homo sapiens cDNA clone IMAGE:40081082. [BC131497]	3,17
PSMB10	NM_002801	ref|Homo sapiens proteasome (prosome, macropain) subunit, beta type, 10 (PSMB10), mRNA [NM_002801]	3,17
NBL1	NM_182744	ref|Homo sapiens neuroblastoma, suppression of tumorigenicity 1 (NBL1), transcript variant 1, mRNA [NM_182744]	3,17
THC2696414	THC2696414	Unknown	3,16
GINS2	NM_016095	ref|Homo sapiens GINS complex subunit 2 (Psf2 homolog) (GINS2), mRNA [NM_016095]	3,16
CTSA	NM_000308	ref|Homo sapiens cathepsin A (CTSA), transcript variant 1, mRNA [NM_000308]	3,16
GM2A	NM_000405	ref|Homo sapiens GM2 ganglioside activator (GM2A), mRNA [NM_000405]	3,16
CHAF1B	NM_005441	ref|Homo sapiens chromatin assembly factor 1, subunit B (p60) (CHAF1B), mRNA [NM_005441]	3,15
AT_ssH_GD_3	AT_ssH_GD_3		3,15
A2M	NM_000014	ref|Homo sapiens alpha-2-macroglobulin (A2M), mRNA [NM_000014]	3,14
TPCN2	NM_139075	ref|Homo sapiens two pore segment channel 2 (TPCN2), mRNA [NM_139075]	3,13
QDPR	M16447	gb|Human dihydropteridine reductase (hDHPR) mRNA, complete cds. [M16447]	3,13
CR618615	CR618615	gb|full-length cDNA clone CL0BB018ZH05 of Neuroblastoma of Homo sapiens (human). [CR618615]	3,13
THC2707492	THC2707492	Unknown	3,12
hCG_1774568	BC004179	gb|Homo sapiens similar to hCG1774568, mRNA (cDNA clone IMAGE:2959703), **** WARNING: chimeric clone ****. [BC004179]	3,11
P2RY2	NM_176072	ref|Homo sapiens purinergic receptor P2Y, G-protein coupled, 2 (P2RY2), transcript variant 1, mRNA [NM_176072]	3,10
DTNA	NM_001392	ref|Homo sapiens dystrobrevin, alpha (DTNA), transcript variant 7, mRNA [NM_001392]	3,09
BDH2	NM_020139	ref|Homo sapiens 3-hydroxybutyrate dehydrogenase, type 2 (BDH2), mRNA [NM_020139]	3,07
CHAF1B	NM_005441	ref|Homo sapiens chromatin assembly factor 1, subunit B (p60) (CHAF1B), mRNA [NM_005441]	3,07
CHAF1B	NM_005441	ref|Homo sapiens chromatin assembly factor 1, subunit B (p60) (CHAF1B), mRNA [NM_005441]	3,07
NAP5	NM_207481	ref|Homo sapiens Nck-associated protein 5 (NAP5), transcript variant 2, mRNA [NM_207481]	3,07
ACP5	NM_001611	ref|Homo sapiens acid phosphatase 5, tartrate resistant (ACP5), transcript variant 4, mRNA [NM_001611]	3,06
CHAF1B	NM_005441	ref|Homo sapiens chromatin assembly factor 1, subunit B (p60) (CHAF1B), mRNA [NM_005441]	3,06
AV749257	AV749257	gb|AV749257 AV749257 NPC Homo sapiens cDNA clone NPCCTB10 5', mRNA sequence [AV749257]	3,06
SPAG4	NM_003116	ref|Homo sapiens sperm associated antigen 4 (SPAG4), mRNA [NM_003116]	3,05
TM4SF19	NM_138461	ref|Homo sapiens transmembrane 4 L six family member 19 (TM4SF19), mRNA [NM_138461]	3,04
BAAT	NM_001701	ref|Homo sapiens bile acid Coenzyme A: amino acid N-acyltransferase (glycine N-choloyltransferase) (BAAT), transcript variant 1, mRNA [NM_001701]	3,04
CHAF1B	NM_005441	ref|Homo sapiens chromatin assembly factor 1, subunit B (p60) (CHAF1B), mRNA [NM_005441]	3,04
PELI3	NM_145065	ref|Homo sapiens pellino homolog 3 (Drosophila) (PELI3), transcript variant 1, mRNA [NM_145065]	3,04
TPK1	NM_022445	ref|Homo sapiens thiamin pyrophosphokinase 1 (TPK1), transcript variant 1, mRNA [NM_022445]	3,03
FCGR2A	NM_021642	ref|Homo sapiens Fc fragment of IgG, low affinity IIa, receptor (CD32) (FCGR2A), transcript variant 2, mRNA [NM_021642]	3,03
CHAF1B	NM_005441	ref|Homo sapiens chromatin assembly factor 1, subunit B (p60) (CHAF1B), mRNA [NM_005441]	3,03
FBLIM1	NM_017556	ref|Homo sapiens filamin binding LIM protein 1 (FBLIM1), transcript variant 1, mRNA [NM_017556]	3,02
CHAF1B	NM_005441	ref|Homo sapiens chromatin assembly factor 1, subunit B (p60) (CHAF1B), mRNA [NM_005441]	3,02
C10orf59	NM_001031709	ref|Homo sapiens chromosome 10 open reading frame 59 (C10orf59), transcript variant 1, mRNA [NM_001031709]	3,02
SPDYA	NM_001008779	ref|Homo sapiens speedy homolog A (Xenopus laevis) (SPDYA), transcript variant 2, mRNA [NM_001008779]	3,02
AK055428	AK055428	gb|Homo sapiens cDNA FLJ30866 fis, clone FEBRA2004110, highly similar to PHOSPHOLIPASE ADRAB-B PRECURSOR (EC 3.1.-.-). [AK055428]	3,02
PDPR	NM_017990	ref|Homo sapiens pyruvate dehydrogenase phosphatase regulatory subunit (PDPR), mRNA [NM_017990]	2,99
LOC388588	NR_024432	ref|Homo sapiens hypothetical LOC388588 (LOC388588), non-coding RNA [NR_024432]	2,99
IMMP2L	NM_032549	ref|Homo sapiens IMP2 inner mitochondrial membrane peptidase-like (S. cerevisiae) (IMMP2L), nuclear gene encoding mitochondrial protein, mRNA [NM_03254	2,99
CHAF1B	NM_005441	ref|Homo sapiens chromatin assembly factor 1, subunit B (p60) (CHAF1B), mRNA [NM_005441]	2,99
MET	NM_000245	ref|Homo sapiens met proto-oncogene (hepatocyte growth factor receptor) (MET), transcript variant 2, mRNA [NM_000245]	2,98
BC008001	BC008001	gb|Homo sapiens mRNA similar to hypothetical protein FLJ21463 (cDNA clone IMAGE:3504595). [BC008001]	2,98
FCGR2B	NM_004001	ref|Homo sapiens Fc fragment of IgG, low affinity IIb, receptor (CD32) (FCGR2B), transcript variant 1, mRNA [NM_004001]	2,98
CHAF1B	NM_005441	ref|Homo sapiens chromatin assembly factor 1, subunit B (p60) (CHAF1B), mRNA [NM_005441]	2,97
MARCH3	NM_178450	ref|Homo sapiens membrane-associated ring finger (C3HC4) 3 (MARCH3), mRNA [NM_178450]	2,97
THC2533854	THC2533854	thc|Q2NUU5_SODGM (Q2NUU5) Glutamate-aspartate ABC transporter ATP-binding component GltL, partial (7%) [THC2533854]	2,97
LAMP3	NM_014398	ref|Homo sapiens lysosomal-associated membrane protein 3 (LAMP3), mRNA [NM_014398]	2,95
NDRG4	NM_022910	ref|Homo sapiens NDRG family member 4 (NDRG4), transcript variant 3, mRNA [NM_022910]	2,95
HIST1H4L	NM_003546	ref|Homo sapiens histone cluster 1, H4l (HIST1H4L), mRNA [NM_003546]	2,95
ADAM23	NM_003812	ref|Homo sapiens ADAM metallopeptidase domain 23 (ADAM23), mRNA [NM_003812]	2,94
LIMCH1	NM_014988	ref|Homo sapiens LIM and calponin homology domains 1 (LIMCH1), transcript variant 1, mRNA [NM_014988]	2,94
NAV2	NM_182964	ref|Homo sapiens neuron navigator 2 (NAV2), transcript variant 1, mRNA [NM_182964]	2,93
TLR4	NM_138554	ref|Homo sapiens toll-like receptor 4 (TLR4), transcript variant 1, mRNA [NM_138554]	2,93
ENST00000372142	ENST00000372142	ens|Pro-neuregulin-3, membrane-bound isoform Precursor (Pro-NRG3) [Contains Neuregulin-3(NRG-3)] [Source:UniProtKB/Swiss-Prot;Acc:P56975] [ENST0000	2,92
AB362555	AB362555	gb|Homo sapiens HLA-DMB mRNA for MHC class II antigen, partial cds, splicing variant 2. [AB362555]	2,91
CHAF1B	NM_005441	ref|Homo sapiens chromatin assembly factor 1, subunit B (p60) (CHAF1B), mRNA [NM_005441]	2,90

TTYH2	NM_032646	ref|Homo sapiens tweety homolog 2 (Drosophila) (TTYH2), transcript variant 1, mRNA [NM_032646]	2,90
TP53TG3	NM_016212	ref|Homo sapiens TP53 target 3 (TP53TG3), mRNA [NM_016212]	2,90
HIST1H4C	NM_003542	ref|Homo sapiens histone cluster 1, H4c (HIST1H4C), mRNA [NM_003542]	2,89
SNCB	NM_001001502	ref|Homo sapiens synuclein, beta (SNCB), transcript variant 1, mRNA [NM_001001502]	2,88
BCL2A1	NM_004049	ref|Homo sapiens BCL2-related protein A1 (BCL2A1), transcript variant 1, mRNA [NM_004049]	2,87
C9orf91	NM_153045	ref|Homo sapiens chromosome 9 open reading frame 91 (C9orf91), mRNA [NM_153045]	2,86
ESPN	NM_031475	ref|Homo sapiens espin (ESPN), mRNA [NM_031475]	2,86
GULP1	NM_016315	ref|Homo sapiens GULP, engulfment adaptor PTB domain containing 1 (GULP1), mRNA [NM_016315]	2,86
ZNF778	NM_182531	ref|Homo sapiens zinc finger protein 778 (ZNF778), mRNA [NM_182531]	2,86
ECOP	NM_030796	ref|Homo sapiens EGFR-coamplified and overexpressed protein (ECOP), mRNA [NM_030796]	2,85
RFX6	NM_173560	ref|Homo sapiens regulatory factor X, 6 (RFX6), mRNA [NM_173560]	2,84
DENND2D	AL713773	Unknown	2,83
MYB	NM_005375	ref|Homo sapiens v-myb myeloblastosis viral oncogene homolog (avian) (MYB), transcript variant 2, mRNA [NM_005375]	2,82
A_24_P127063	A_24_P127063	Unknown	2,81
CREB3L2	BC063666	gb|Homo sapiens cAMP responsive element binding protein 3-like 2, mRNA (cDNA clone IMAGE:4185677), complete cds. [BC063666]	2,79
DQ786230	DQ786230	gb|Homo sapiens clone HLS_IMAGE_1706664 mRNA sequence. [DQ786230]	2,77
RASSF4	NM_032023	ref|Homo sapiens Ras association (RalGDS/AF-6) domain family member 4 (RASSF4), mRNA [NM_032023]	2,77
GRAP	NM_006613	ref|Homo sapiens GRB2-related adaptor protein (GRAP), mRNA [NM_006613]	2,77
FGGY	NM_018291	ref|Homo sapiens FGGY carbohydrate kinase domain containing (FGGY), transcript variant 2, mRNA [NM_018291]	2,75
STARD8	NM_014725	ref|Homo sapiens StAR-related lipid transfer (START) domain containing 8 (STARD8), transcript variant 2, mRNA [NM_014725]	2,75
SNN	NM_003498	ref|Homo sapiens stannin (SNN), mRNA [NM_003498]	2,75
TRPM6	NM_017662	ref|Homo sapiens transient receptor potential cation channel, subfamily M, member 6 (TRPM6), mRNA [NM_017662]	2,74
GLB1L3	NM_001080407	ref|Homo sapiens galactosidase, beta 1-like 3 (GLB1L3), mRNA [NM_001080407]	2,74
PXMP2	NM_018663	ref|Homo sapiens peroxisomal membrane protein 2, 22kDa (PXMP2), mRNA [NM_018663]	2,73
SLC37A2	NM_198277	ref|Homo sapiens solute carrier family 37 (glycerol-3-phosphate transporter), member 2 (SLC37A2), mRNA [NM_198277]	2,72
CCR7	NM_001838	ref|Homo sapiens chemokine (C-C motif) receptor 7 (CCR7), mRNA [NM_001838]	2,71
A_32_P93792	A_32_P93792	Unknown	2,71
OLFM1	NM_006334	ref|Homo sapiens olfactomedin 1 (OLFM1), transcript variant 2, mRNA [NM_006334]	2,70
CACNA1G	NM_018896	ref|Homo sapiens calcium channel, voltage-dependent, T type, alpha 1G subunit (CACNA1G), transcript variant 1, mRNA [NM_018896]	2,70
CHCHD7	NM_001011667	ref|Homo sapiens coiled-coil-helix-coiled-coil-helix domain containing 7 (CHCHD7), transcript variant 1, mRNA [NM_001011667]	2,70
C5orf47	ENST00000340147	ens|Uncharacterized protein C5orf47 [Source:UniProtKB/Swiss-Prot;Acc:Q569G3] [ENST00000340147]	2,70
SLC5A4	NM_014227	ref|Homo sapiens solute carrier family 5 (low affinity glucose cotransporter), member 4 (SLC5A4), mRNA [NM_014227]	2,70
TMCC3	NM_020698	ref|Homo sapiens transmembrane and coiled-coil domain family 3 (TMCC3), mRNA [NM_020698]	2,70
THC2750781	THC2750781	Unknown	2,69
CTSA	NM_000308	ref|Homo sapiens cathepsin A (CTSA), transcript variant 1, mRNA [NM_000308]	2,68
ACN9	NM_020186	ref|Homo sapiens ACN9 homolog (S. cerevisiae) (ACN9), mRNA [NM_020186]	2,65
WISP2	NM_003881	ref|Homo sapiens WNT1 inducible signaling pathway protein 2 (WISP2), mRNA [NM_003881]	2,65
DHRS13	NM_144683	ref|Homo sapiens dehydrogenase/reductase (SDR family) member 13 (DHRS13), mRNA [NM_144683]	2,65
TP53TG3	NM_016212	ref|Homo sapiens TP53 target 3 (TP53TG3), mRNA [NM_016212]	2,64
OXNAD1	NM_138381	ref|Homo sapiens oxidoreductase NAD-binding domain containing 1 (OXNAD1), mRNA [NM_138381]	2,63
BC015977	BC015977	gb|Homo sapiens, clone IMAGE:4042121, mRNA, partial cds. [BC015977]	2,61
MGC16025	BC008026	gb|Homo sapiens hypothetical protein MGC16025, mRNA (cDNA clone MGC:16025 IMAGE:3607793), complete cds. [BC008026]	2,61
RAD51L1	NM_133510	ref|Homo sapiens RAD51-like 1 (S. cerevisiae) (RAD51L1), transcript variant 2, mRNA [NM_133510]	2,58
RBM33	BC011923	gb|Homo sapiens RNA binding motif protein 33, mRNA (cDNA clone IMAGE:4542314), complete cds. [BC011923]	2,52
