# Supplementary material for: MITF-Independent Pro-Survival Role of BRG1-Containing SWI/SNF Complex in Melanoma Cells
Source: PLoS One. 2013 Jan 17;8(1):e54110. doi: 10.1371/journal.pone.0054110 (PMC3547967; doi:10.1371/journal.pone.0054110)
Supplement: Table S2 — Known MITF target genes downregulated more than 2-fold by BRG1 knockdown in 501mel cells (P<0.005). (MITF itself was downregulated ∼5-fold). (PDF) [file pone.0054110.s005.pdf]

Supplementary Table 2. Known MITF target genes dowregulated more than 2-fold by Brg1 knockdown in 501mel cells (P<0.005). (MITF itself was downregulated ~5-fold).

Gene symbol	Gene name	Genebank	-fold
DCT	dopachrome tautomerase (dopachrome delta-isomerase, tyrosine-related protein 2) (DCT),transcript variant 1, mRNA	NM_001922	22.50
TRPM1	transient receptor potential cation channel, subfamily M, member 1 (TRPM1), mRNA	NM_002420	15.98
MLANA	melan-A (MLANA), mRNA	NM_005511	10.76
RAB27A	RAB27A, member RAS oncogene family (RAB27A), transcript variant 1, mRNA	NM_004580	6.72
BIRC7	baculoviral IAP repeat-containing 7 (BIRC7), transcript variant 2, mRNA	NM_022161	5.85
CDK2	cyclin-dependent kinase 2 (CDK2), transcript variant 1, mRNA	NM_001798	4.98
TYRP1	tyrosinase-related protein 1 (TYRP1), mRNA	NM_000550	4.98
SILV	silver homolog (mouse) (SILV), mRNA	NM_006928	4.21
SLC45A2	solute carrier family 45, member 2 (SLC45A2), transcript variant 1, mRNA	NM_016180	4.12
SNAI2	snail homolog 2 (Drosophila) (SNAI2), mRNA	NM_003068	3,76
TYR	tyrosinase (oculocutaneous albinism IA) (TYR), mRNA	NM_000372	3.49
GPR143	G protein-coupled receptor 143 (GPR143), mRNA	NM_000273	3.27
MET	met proto-oncogene (hepatocyte growth factor receptor) (MET), transcript variant 2, mRNA	NM_000245	2.98
BCL2	B-cell CLL/lymphoma 2 (BCL2), nuclear gene encoding mitochondrial protein, transcript variant alpha, mRNA	NM_000633	2.38
EDNRB	endothelin receptor type B (EDNRB), transcript variant 2, mRNA	NM_003991	2.23
