# Supplementary material for: MITF-Independent Pro-Survival Role of BRG1-Containing SWI/SNF Complex in Melanoma Cells
Source: PLoS One. 2013 Jan 17;8(1):e54110. doi: 10.1371/journal.pone.0054110 (PMC3547967; doi:10.1371/journal.pone.0054110)
Supplement: Table S3 — Microarray analysis of genes up-regulated >2-fold (P<0.005) by BRG1 depletion in 501mel cells. (PDF) [file pone.0054110.s006.pdf]

Supplementary Table S3. Microarray analysis of genes up-regulated >2-fold (P<0.005) by Brg1 depletion in 501mel cells.

Gene Symbol	Systematic Name	Gene Title	Fold change
COL13A1	NM_005203	ref|Homo sapiens collagen, type XIII, alpha 1 (COL13A1), transcript variant 1, mRNA [NM_005203]	23,203
COL13A1	NM_005203	ref|Homo sapiens collagen, type XIII, alpha 1 (COL13A1), transcript variant 1, mRNA [NM_005203]	20,793
COL13A1	NM_005203	ref|Homo sapiens collagen, type XIII, alpha 1 (COL13A1), transcript variant 1, mRNA [NM_005203]	20,769
COL13A1	NM_005203	ref|Homo sapiens collagen, type XIII, alpha 1 (COL13A1), transcript variant 1, mRNA [NM_005203]	19,676
COL13A1	NM_005203	ref|Homo sapiens collagen, type XIII, alpha 1 (COL13A1), transcript variant 1, mRNA [NM_005203]	19,074
NPTX1	NM_002522	ref|Homo sapiens neuronal pentraxin I (NPTX1), mRNA [NM_002522]	18,548
COL13A1	NM_005203	ref|Homo sapiens collagen, type XIII, alpha 1 (COL13A1), transcript variant 1, mRNA [NM_005203]	18,126
HMGCS1	NM_002130	ref|Homo sapiens 3-hydroxy-3-methylglutaryl-Coenzyme A synthase 1 (soluble) (HMGCS1), transcript variant 2, mRNA [NM_002	15,489
COL13A1	NM_005203	ref|Homo sapiens collagen, type XIII, alpha 1 (COL13A1), transcript variant 1, mRNA [NM_005203]	14,214
COL13A1	NM_005203	ref|Homo sapiens collagen, type XIII, alpha 1 (COL13A1), transcript variant 1, mRNA [NM_005203]	14,073
CXCL14	NM_004887	ref|Homo sapiens chemokine (C-X-C motif) ligand 14 (CXCL14), mRNA [NM_004887]	13,918
COL13A1	NM_005203	ref|Homo sapiens collagen, type XIII, alpha 1 (COL13A1), transcript variant 1, mRNA [NM_005203]	12,630
COL13A1	NM_005203	ref|Homo sapiens collagen, type XIII, alpha 1 (COL13A1), transcript variant 1, mRNA [NM_005203]	11,752
KRT34	NM_021013	ref|Homo sapiens keratin 34 (KRT34), mRNA [NM_021013]	11,283
COL13A1	NM_005203	ref|Homo sapiens collagen, type XIII, alpha 1 (COL13A1), transcript variant 1, mRNA [NM_005203]	10,367
HMGCS1	NM_002130	ref|Homo sapiens 3-hydroxy-3-methylglutaryl-Coenzyme A synthase 1 (soluble) (HMGCS1), transcript variant 2, mRNA [NM_002	10,278
ITGA1	NM_181501	ref|Homo sapiens integrin, alpha 1 (ITGA1), mRNA [NM_181501]	10,271
KCNN4	NM_002250	ref|Homo sapiens potassium intermediate/small conductance calcium-activated channel, subfamily N, member 4 (KCNN4), mRN	9,800
THC2681410	THC2681410	Unknown	9,550
HIST2H2AA4	NM_001040874	ref|Homo sapiens histone cluster 2, H2aa4 (HIST2H2AA4), mRNA [NM_001040874]	9,185
HIST2H2AA4	NM_001040874	ref|Homo sapiens histone cluster 2, H2aa4 (HIST2H2AA4), mRNA [NM_001040874]	8,980
HSD17B7P2	AF165514	gb|Homo sapiens 17-beta-hydroxysteroid dehydrogenase type VII isoform mRNA, complete cds. [AF165514]	8,211
ACAT2	NM_005891	ref|Homo sapiens acetyl-Coenzyme A acetyltransferase 2 (ACAT2), mRNA [NM_005891]	8,175
A_23_P147404	A_23_P147404	Unknown	7,967
ACAT2	NM_005891	ref|Homo sapiens acetyl-Coenzyme A acetyltransferase 2 (ACAT2), mRNA [NM_005891]	7,966
LSS	NM_001001438	ref|Homo sapiens lanosterol synthase (2,3-oxidosqualene-lanosterol cyclase) (LSS), transcript variant 2, mRNA [NM_001001438	7,672
NPTX1	NM_002522	ref|Homo sapiens neuronal pentraxin I (NPTX1), mRNA [NM_002522]	7,658
HSD17B7	NM_016371	ref|Homo sapiens hydroxysteroid (17-beta) dehydrogenase 7 (HSD17B7), mRNA [NM_016371]	7,635
HSD17B7	NM_016371	ref|Homo sapiens hydroxysteroid (17-beta) dehydrogenase 7 (HSD17B7), mRNA [NM_016371]	7,312
IDI1	NM_004508	ref|Homo sapiens isopentenyl-diphosphate delta isomerase 1 (IDI1), mRNA [NM_004508]	7,103
MST150	NM_032947	ref|Homo sapiens MSTP150 (MST150), mRNA [NM_032947]	7,028
DKK1	NM_012242	ref|Homo sapiens dickkopf homolog 1 (Xenopus laevis) (DKK1), mRNA [NM_012242]	7,023
HIST1H2AD	NM_021065	ref|Homo sapiens histone cluster 1, H2ad (HIST1H2AD), mRNA [NM_021065]	6,921
TMEM158	NM_015444	ref|Homo sapiens transmembrane protein 158 (TMEM158), mRNA [NM_015444]	6,886
LDLR	NM_000527	ref|Homo sapiens low density lipoprotein receptor (LDLR), mRNA [NM_000527]	6,476
IL3RA	NM_002183	ref|Homo sapiens interleukin 3 receptor, alpha (low affinity) (IL3RA), mRNA [NM_002183]	6,409
IL21R	NM_181078	ref|Homo sapiens interleukin 21 receptor (IL21R), transcript variant 2, mRNA [NM_181078]	6,344
LSS	NM_001001438	ref|Homo sapiens lanosterol synthase (2,3-oxidosqualene-lanosterol cyclase) (LSS), transcript variant 2, mRNA [NM_001001438	6,340
HIST1H1C	NM_005319	ref|Homo sapiens histone cluster 1, H1c (HIST1H1C), mRNA [NM_005319]	6,301
EGR4	NM_001965	ref|Homo sapiens early growth response 4 (EGR4), mRNA [NM_001965]	6,293
HIST2H2AB	NM_175065	ref|Homo sapiens histone cluster 2, H2ab (HIST2H2AB), mRNA [NM_175065]	6,230
LRRN3	NM_018334	ref|Homo sapiens leucine rich repeat neuronal 3 (LRRN3), transcript variant 3, mRNA [NM_018334]	6,210
SC4MOL	NM_006745	ref|Homo sapiens sterol-C4-methyl oxidase-like (SC4MOL), transcript variant 1, mRNA [NM_006745]	6,173
LDLR	NM_000527	ref|Homo sapiens low density lipoprotein receptor (LDLR), mRNA [NM_000527]	6,126
CADPS	NM_183393	ref|Homo sapiens Ca++-dependent secretion activator (CADPS), transcript variant 3, mRNA [NM_183393]	6,054
CDKN2B	NM_078487	ref|Homo sapiens cyclin-dependent kinase inhibitor 2B (p15, inhibits CDK4) (CDKN2B), transcript variant 2, mRNA [NM_078487]	6,004
NSDHL	NM_015922	ref|Homo sapiens NAD(P) dependent steroid dehydrogenase-like (NSDHL), transcript variant 1, mRNA [NM_015922]	5,936
AW993939	AW993939	gb|AW993939 RC1-BN0035-130400-013-b06 BN0035 Homo sapiens cDNA, mRNA sequence [AW993939]	5,921
CR603951	CR603951	gb|full-length cDNA clone CS0DM011YC22 of Fetal liver of Homo sapiens (human). [CR603951]	5,865
FDPS	NM_002004	ref|Homo sapiens farnesyl diphosphate synthase (farnesyl pyrophosphate synthetase, dimethylallyltranstransferase, geranyltrans	5,800
UNC5B	NM_170744	ref|Homo sapiens unc-5 homolog B (C. elegans) (UNC5B), mRNA [NM_170744]	5,763
PPFIBP1	NM_003622	ref|Homo sapiens PTPRF interacting protein, binding protein 1 (liprin beta 1) (PPFIBP1), transcript variant 1, mRNA [NM_003622	5,756
A_23_P170713	A_23_P170713	Unknown	5,751
ACSS2	NM_018677	ref|Homo sapiens acyl-CoA synthetase short-chain family member 2 (ACSS2), transcript variant 1, mRNA [NM_018677]	5,729
HIST1H2AE	NM_021052	ref|Homo sapiens histone cluster 1, H2ae (HIST1H2AE), mRNA [NM_021052]	5,681
ADAM19	NM_033274	ref|Homo sapiens ADAM metallopeptidase domain 19 (meltrin beta) (ADAM19), transcript variant 2, mRNA [NM_033274]	5,681
GZMA	NM_006144	ref|Homo sapiens granzyme A (granzyme 1, cytotoxic T-lymphocyte-associated serine esterase 3) (GZMA), mRNA [NM_006144]	5,661
SLC1A3	NM_004172	ref|Homo sapiens solute carrier family 1 (glial high affinity glutamate transporter), member 3 (SLC1A3), mRNA [NM_004172]	5,641
X15674	X15674	gb|Human pTR5 mRNA for repetitive sequence. [X15674]	5,571
FDPSL2A	NR_003262	ref|Homo sapiens MGC44478 (FDPSL2A), non-coding RNA [NR_003262]	5,566
PPFIBP1	NM_003622	ref|Homo sapiens PTPRF interacting protein, binding protein 1 (liprin beta 1) (PPFIBP1), transcript variant 1, mRNA [NM_003622	5,518
INSIG1	NM_198336	ref|Homo sapiens insulin induced gene 1 (INSIG1), transcript variant 2, mRNA [NM_198336]	5,457
MVK	NM_000431	ref|Homo sapiens mevalonate kinase (MVK), transcript variant 1, mRNA [NM_000431]	5,366
VWCE	NM_152718	ref|Homo sapiens von Willebrand factor C and EGF domains (VWCE), mRNA [NM_152718]	5,356
HIST2H2AC	NM_003517	ref|Homo sapiens histone cluster 2, H2ac (HIST2H2AC), mRNA [NM_003517]	5,303
PPFIBP1	NM_003622	ref|Homo sapiens PTPRF interacting protein, binding protein 1 (liprin beta 1) (PPFIBP1), transcript variant 1, mRNA [NM_003622	5,295
PMEPA1	NM_020182	ref|Homo sapiens prostate transmembrane protein, androgen induced 1 (PMEPA1), transcript variant 1, mRNA [NM_020182]	5,225
A_24_P67268	A_24_P67268	Unknown	5,225
THC2523793	THC2523793	thc|Q9DC07_MOUSE (Q9DC07) Adult male lung cDNA, RIKEN full-length enriched library, clone:1200007O21 product:LIM AND	5,216
BMP6	NM_001718	ref|Homo sapiens bone morphogenetic protein 6 (BMP6), mRNA [NM_001718]	5,082
TGFBI	NM_000358	ref|Homo sapiens transforming growth factor, beta-induced, 68kDa (TGFBI), mRNA [NM_000358]	5,081
ACSS2	NM_018677	ref|Homo sapiens acyl-CoA synthetase short-chain family member 2 (ACSS2), transcript variant 1, mRNA [NM_018677]	5,055
ANKRD36B	AK024934	gb|Homo sapiens cDNA: FLJ21281 fis, clone COL01904. [AK024934]	5,019
RIMS3	NM_014747	ref|Homo sapiens regulating synaptic membrane exocytosis 3 (RIMS3), mRNA [NM_014747]	5,010
CDKN2B	NM_004936	ref|Homo sapiens cyclin-dependent kinase inhibitor 2B (p15, inhibits CDK4) (CDKN2B), transcript variant 1, mRNA [NM_004936]	4,996
DHCR7	NM_001360	ref|Homo sapiens 7-dehydrocholesterol reductase (DHCR7), mRNA [NM_001360]	4,985
CXCL1	NM_001511	ref|Homo sapiens chemokine (C-X-C motif) ligand 1 (melanoma growth stimulating activity, alpha) (CXCL1), mRNA [NM_001511	4,843
EIF4G3	NM_003760	ref|Homo sapiens eukaryotic translation initiation factor 4 gamma, 3 (EIF4G3), mRNA [NM_003760]	4,832
TMEM55B	NM_144568	ref|Homo sapiens transmembrane protein 55B (TMEM55B), transcript variant 2, mRNA [NM_144568]	4,810
IQCD	NM_138451	ref|Homo sapiens IQ motif containing D (IQCD), mRNA [NM_138451]	4,805
FDFT1	NM_004462	ref|Homo sapiens farnesyl-diphosphate farnesyltransferase 1 (FDFT1), mRNA [NM_004462]	4,688
PLAC2	BC036545	gb|Homo sapiens placenta-specific 2 (non-protein coding), mRNA (cDNA clone MGC:43133 IMAGE:5261222), complete cds. [B(	4,656
USP36	AK022913	gb|Homo sapiens cDNA FLJ12851 fis, clone NT2RP2003401, weakly similar to UBIQUITIN CARBOXYL-TERMINAL HYDROLAS	4,628
KIAA1683	NM_025249	ref|Homo sapiens KIAA1683 (KIAA1683), mRNA [NM_025249]	4,620
ATP2A1	NM_173201	ref|Homo sapiens ATPase, Ca++ transporting, cardiac muscle, fast twitch 1 (ATP2A1), transcript variant a, mRNA [NM_173201]	4,619
JAM2	NM_021219	ref|Homo sapiens junctional adhesion molecule 2 (JAM2), mRNA [NM_021219]	4,608
WDR37	NM_014023	ref|Homo sapiens WD repeat domain 37 (WDR37), mRNA [NM_014023]	4,580
CYR61	NM_001554	ref|Homo sapiens cysteine-rich, angiogenic inducer, 61 (CYR61), mRNA [NM_001554]	4,578
HMGCR	NM_000859	ref|Homo sapiens 3-hydroxy-3-methylglutaryl-Coenzyme A reductase (HMGCR), transcript variant 1, mRNA [NM_000859]	4,562
DOCK4	NM_014705	ref|Homo sapiens dedicator of cytokinesis 4 (DOCK4), mRNA [NM_014705]	4,554
ANKRD23	NM_144994	ref|Homo sapiens ankyrin repeat domain 23 (ANKRD23), mRNA [NM_144994]	4,512
C14orf1	NM_007176	ref|Homo sapiens chromosome 14 open reading frame 1 (C14orf1), mRNA [NM_007176]	4,484
FAM105A	NM_019018	ref|Homo sapiens family with sequence similarity 105, member A (FAM105A), mRNA [NM_019018]	4,457
MVK	NM_000431	ref|Homo sapiens mevalonate kinase (MVK), transcript variant 1, mRNA [NM_000431]	4,452
A_24_P834646	A_24_P834646	Unknown	4,450
SQLE	NM_003129	ref|Homo sapiens squalene epoxidase (SQLE), mRNA [NM_003129]	4,449
MVD	NM_002461	ref|Homo sapiens mevalonate (diphospho) decarboxylase (MVD), mRNA [NM_002461]	4,423
LMO2	NM_005574	ref|Homo sapiens LIM domain only 2 (rhombotin-like 1) (LMO2), transcript variant 1, mRNA [NM_005574]	4,357
ASGR1	NM_001671	ref|Homo sapiens asialoglycoprotein receptor 1 (ASGR1), mRNA [NM_001671]	4,330
LMO2	NM_005574	ref|Homo sapiens LIM domain only 2 (rhombotin-like 1) (LMO2), transcript variant 1, mRNA [NM_005574]	4,325
IL22RA1	NM_021258	ref|Homo sapiens interleukin 22 receptor, alpha 1 (IL22RA1), mRNA [NM_021258]	4,274
PEG10	NM_001040152	ref|Homo sapiens paternally expressed 10 (PEG10), transcript variant 1, mRNA [NM_001040152]	4,257

MGC23284	NR_024399	ref|Homo sapiens hypothetical LOC197187 (MGC23284), transcript variant 2, non-coding RNA [NR_024399]	4,252
DERL3	NM_198440	ref|Homo sapiens Der1-like domain family, member 3 (DERL3), transcript variant 3, mRNA [NM_198440]	4,215
THC2655811	THC2655811	Unknown	4,166
PRDM2	NM_012231	ref|Homo sapiens PR domain containing 2, with ZNF domain (PRDM2), transcript variant 1, mRNA [NM_012231]	4,164
ITPKA	NM_002220	ref|Homo sapiens inositol 1,4,5-trisphosphate 3-kinase A (ITPKA), mRNA [NM_002220]	4,132
CYR61	NM_001554	ref|Homo sapiens cysteine-rich, angiogenic inducer, 61 (CYR61), mRNA [NM_001554]	4,115
WNT11	NM_004626	ref|Homo sapiens wingless-type MMTV integration site family, member 11 (WNT11), mRNA [NM_004626]	4,096
UNC5CL	NM_173561	ref|Homo sapiens unc-5 homolog C (C. elegans)-like (UNC5CL), mRNA [NM_173561]	4,065
CYP51A1	NM_000786	ref|Homo sapiens cytochrome P450, family 51, subfamily A, polypeptide 1 (CYP51A1), mRNA [NM_000786]	4,057
PIK3IP1	NM_052880	ref|Homo sapiens phosphoinositide-3-kinase interacting protein 1 (PIK3IP1), transcript variant 1, mRNA [NM_052880]	4,009
IGDCC3	NM_004884	ref|Homo sapiens immunoglobulin superfamily, DCC subclass, member 3 (IGDCC3), mRNA [NM_004884]	3,990
HIST2H2BE	NM_003528	ref|Homo sapiens histone cluster 2, H2be (HIST2H2BE), mRNA [NM_003528]	3,982
CYB5B	NM_030579	ref|Homo sapiens cytochrome b5 type B (outer mitochondrial membrane) (CYB5B), nuclear gene encoding mitochondrial protein	3,949
ZNF720	NM_001130913	ref|Homo sapiens zinc finger protein 720 (ZNF720), mRNA [NM_001130913]	3,924
HIST1H1D	NM_005320	ref|Homo sapiens histone cluster 1, H1d (HIST1H1D), mRNA [NM_005320]	3,905
MMAB	NM_052845	ref|Homo sapiens methylmalonic aciduria (cobalamin deficiency) cblB type (MMAB), nuclear gene encoding mitochondrial protein	3,890
LPIN1	NM_145693	ref|Homo sapiens lipin 1 (LPIN1), mRNA [NM_145693]	3,873
SCARA5	NM_173833	ref|Homo sapiens scavenger receptor class A, member 5 (putative) (SCARA5), mRNA [NM_173833]	3,867
TM7SF2	NM_003273	ref|Homo sapiens transmembrane 7 superfamily member 2 (TM7SF2), mRNA [NM_003273]	3,862
OVGP1	NM_002557	ref|Homo sapiens oviductal glycoprotein 1, 120kDa (OVGP1), mRNA [NM_002557]	3,815
SERPINB8	NM_001031848	ref|Homo sapiens serpin peptidase inhibitor, clade B (ovalbumin), member 8 (SERPINB8), transcript variant 3, mRNA [NM_0010	3,812
LMO2	NM_005574	ref|Homo sapiens LIM domain only 2 (rhombotin-like 1) (LMO2), transcript variant 1, mRNA [NM_005574]	3,807
LMO2	NM_005574	ref|Homo sapiens LIM domain only 2 (rhombotin-like 1) (LMO2), transcript variant 1, mRNA [NM_005574]	3,807
BDNF	NM_170735	ref|Homo sapiens brain-derived neurotrophic factor (BDNF), transcript variant 1, mRNA [NM_170735]	3,796
TM9SF4	NM_014742	ref|Homo sapiens transmembrane 9 superfamily protein member 4 (TM9SF4), mRNA [NM_014742]	3,788
TESC	NM_017899	ref|Homo sapiens tescalcin (TESC), mRNA [NM_017899]	3,779
A_24_P196134	A_24_P196134	Unknown	3,777
CYP51A1	NM_000786	ref|Homo sapiens cytochrome P450, family 51, subfamily A, polypeptide 1 (CYP51A1), mRNA [NM_000786]	3,762
JUN	NM_002228	ref|Homo sapiens jun oncogene (JUN), mRNA [NM_002228]	3,762
CYR61	NM_001554	ref|Homo sapiens cysteine-rich, angiogenic inducer, 61 (CYR61), mRNA [NM_001554]	3,754
SSTR2	NM_001050	ref|Homo sapiens somatostatin receptor 2 (SSTR2), mRNA [NM_001050]	3,750
JUN	NM_002228	ref|Homo sapiens jun oncogene (JUN), mRNA [NM_002228]	3,744
PTGS2	NM_000963	ref|Homo sapiens prostaglandin-endoperoxide synthase 2 (prostaglandin G/H synthase and cyclooxygenase) (PTGS2), mRNA [N	3,741
DNAJB6	NM_058246	ref|Homo sapiens DnaJ (Hsp40) homolog, subfamily B, member 6 (DNAJB6), transcript variant 1, mRNA [NM_058246]	3,732
MMAB	NM_052845	ref|Homo sapiens methylmalonic aciduria (cobalamin deficiency) cblB type (MMAB), nuclear gene encoding mitochondrial protein	3,729
JUN	NM_002228	ref|Homo sapiens jun oncogene (JUN), mRNA [NM_002228]	3,722
THC2665398	THC2665398	thc|Q5T0S6_HUMAN (Q5T0S6) Palmitoyl-protein thioesterase 1 (Ceroid-lipofuscinosis, neuronal 1, infantile) (Fragment), partial	3,705
SYNPO2	NM_001128933	ref|Homo sapiens synaptopodin 2 (SYNPO2), transcript variant 2, mRNA [NM_001128933]	3,703
TDO2	NM_005651	ref|Homo sapiens tryptophan 2,3-dioxygenase (TDO2), mRNA [NM_005651]	3,700
ARMC9	NM_025139	ref|Homo sapiens armadillo repeat containing 9 (ARMC9), mRNA [NM_025139]	3,699
LOC150759	AK057596	gb|Homo sapiens cDNA FLJ33034 fis, clone THYMU2000236. [AK057596]	3,660
PTGS2	NM_000963	ref|Homo sapiens prostaglandin-endoperoxide synthase 2 (prostaglandin G/H synthase and cyclooxygenase) (PTGS2), mRNA [N	3,656
YPEL2	NM_001005404	ref|Homo sapiens yippee-like 2 (Drosophila) (YPEL2), mRNA [NM_001005404]	3,654
JUN	NM_002228	ref|Homo sapiens jun oncogene (JUN), mRNA [NM_002228]	3,628
JUN	NM_002228	ref|Homo sapiens jun oncogene (JUN), mRNA [NM_002228]	3,613
LMO2	NM_005574	ref|Homo sapiens LIM domain only 2 (rhombotin-like 1) (LMO2), transcript variant 1, mRNA [NM_005574]	3,589
SLC35F1	NM_001029858	ref|Homo sapiens solute carrier family 35, member F1 (SLC35F1), mRNA [NM_001029858]	3,586
NR2F1	NM_005654	ref|Homo sapiens nuclear receptor subfamily 2, group F, member 1 (NR2F1), mRNA [NM_005654]	3,578
THC2577566	THC2577566	Unknown	3,572
JUN	NM_002228	ref|Homo sapiens jun oncogene (JUN), mRNA [NM_002228]	3,560
PPFIBP1	NM_177444	ref|Homo sapiens PTPRF interacting protein, binding protein 1 (liprin beta 1) (PPFIBP1), transcript variant 2, mRNA [NM_177444	3,530
PTGS2	NM_000963	ref|Homo sapiens prostaglandin-endoperoxide synthase 2 (prostaglandin G/H synthase and cyclooxygenase) (PTGS2), mRNA [N	3,518
JUN	NM_002228	ref|Homo sapiens jun oncogene (JUN), mRNA [NM_002228]	3,517
JUN	NM_002228	ref|Homo sapiens jun oncogene (JUN), mRNA [NM_002228]	3,502
WFIKKN1	NM_053284	ref|Homo sapiens WAP, follistatin/kazal, immunoglobulin, kunitz and netrin domain containing 1 (WFIKKN1), mRNA [NM_053284	3,486
JUN	NM_002228	ref|Homo sapiens jun oncogene (JUN), mRNA [NM_002228]	3,478
JUN	NM_002228	ref|Homo sapiens jun oncogene (JUN), mRNA [NM_002228]	3,463
ZNF425	NM_001001661	ref|Homo sapiens zinc finger protein 425 (ZNF425), mRNA [NM_001001661]	3,461
HBP1	NM_012257	ref|Homo sapiens HMG-box transcription factor 1 (HBP1), mRNA [NM_012257]	3,461
MAP2	NM_002374	ref|Homo sapiens microtubule-associated protein 2 (MAP2), transcript variant 1, mRNA [NM_002374]	3,461
BASP1	NM_006317	ref|Homo sapiens brain abundant, membrane attached signal protein 1 (BASP1), mRNA [NM_006317]	3,459
AF087999	AF087999	gb|Homo sapiens full length insert cDNA clone YX44E03. [AF087999]	3,452
GPR35	NM_005301	ref|Homo sapiens G protein-coupled receptor 35 (GPR35), mRNA [NM_005301]	3,451
ZNF75D	NM_007131	ref|Homo sapiens zinc finger protein 75D (ZNF75D), mRNA [NM_007131]	3,434
AOC3	NM_003734	ref|Homo sapiens amine oxidase, copper containing 3 (vascular adhesion protein 1) (AOC3), mRNA [NM_003734]	3,424
ZNF264	NM_003417	ref|Homo sapiens zinc finger protein 264 (ZNF264), mRNA [NM_003417]	3,417
TMEM97	NM_014573	ref|Homo sapiens transmembrane protein 97 (TMEM97), mRNA [NM_014573]	3,412
TP53	NM_000546	ref|Homo sapiens tumor protein p53 (TP53), transcript variant 1, mRNA [NM_000546]	3,403
TP53	NM_000546	ref|Homo sapiens tumor protein p53 (TP53), transcript variant 1, mRNA [NM_000546]	3,396
LMO2	NM_005574	ref|Homo sapiens LIM domain only 2 (rhombotin-like 1) (LMO2), transcript variant 1, mRNA [NM_005574]	3,393
LMO2	NM_005574	ref|Homo sapiens LIM domain only 2 (rhombotin-like 1) (LMO2), transcript variant 1, mRNA [NM_005574]	3,388
MXD1	NM_002357	ref|Homo sapiens MAX dimerization protein 1 (MXD1), mRNA [NM_002357]	3,365
YPEL2	NM_001005404	ref|Homo sapiens yippee-like 2 (Drosophila) (YPEL2), mRNA [NM_001005404]	3,351
CTSK	NM_000396	ref|Homo sapiens cathepsin K (CTSK), mRNA [NM_000396]	3,350
TMEM97	NM_014573	ref|Homo sapiens transmembrane protein 97 (TMEM97), mRNA [NM_014573]	3,337
TMEM97	NM_014573	ref|Homo sapiens transmembrane protein 97 (TMEM97), mRNA [NM_014573]	3,335
DOCK4	NM_014705	ref|Homo sapiens dedicator of cytokinesis 4 (DOCK4), mRNA [NM_014705]	3,333
RNF20	NM_019592	ref|Homo sapiens ring finger protein 20 (RNF20), mRNA [NM_019592]	3,324
HIST1H2AC	ENST00000314088	ens|Histone H2A type 1-C (H2A/l) [Source:UniProtKB/Swiss-Prot;Acc:Q93077] [ENST00000314088]	3,302
RDH16	NM_003708	ref|Homo sapiens retinol dehydrogenase 16 (all-trans) (RDH16), mRNA [NM_003708]	3,301
SC5DL	NM_001024956	ref|Homo sapiens sterol-C5-desaturase (ERG3 delta-5-desaturase homolog, S. cerevisiae)-like (SC5DL), transcript variant 2, mR	3,292
GNG11	NM_004126	ref|Homo sapiens guanine nucleotide binding protein (G protein), gamma 11 (GNG11), mRNA [NM_004126]	3,290
PLAUR	NM_001005377	ref|Homo sapiens plasminogen activator, urokinase receptor (PLAUR), transcript variant 3, mRNA [NM_001005377]	3,273
GREM2	NM_022469	ref|Homo sapiens gremlin 2, cysteine knot superfamily, homolog (Xenopus laevis) (GREM2), mRNA [NM_022469]	3,273
PTGER4	NM_000958	ref|Homo sapiens prostaglandin E receptor 4 (subtype EP4) (PTGER4), mRNA [NM_000958]	3,271
IL8	NM_000584	ref|Homo sapiens interleukin 8 (IL8), mRNA [NM_000584]	3,264
LMO2	NM_005574	ref|Homo sapiens LIM domain only 2 (rhombotin-like 1) (LMO2), transcript variant 1, mRNA [NM_005574]	3,261
TMEM81	NM_203376	ref|Homo sapiens transmembrane protein 81 (TMEM81), mRNA [NM_203376]	3,253
FRG2	NM_001005217	ref|Homo sapiens FSHD region gene 2 (FRG2), mRNA [NM_001005217]	3,236
BX107298	BX107298	gb|BX107298 Soares_testis_NHT Homo sapiens cDNA clone IMAGp998N011789, mRNA sequence [BX107298]	3,227
TFPI	NM_006287	ref|Homo sapiens tissue factor pathway inhibitor (lipoprotein-associated coagulation inhibitor) (TFPI), transcript variant 1, mRNA	3,183
ADAM8	NM_001109	ref|Homo sapiens ADAM metallopeptidase domain 8 (ADAM8), mRNA [NM_001109]	3,179
CR596670	CR596670	gb|full-length cDNA clone CS0DF018YM18 of Fetal brain of Homo sapiens (human). [CR596670]	3,173
CLCN6	NM_001286	ref|Homo sapiens chloride channel 6 (CLCN6), transcript variant ClC-6a, mRNA [NM_001286]	3,171
ERO1LB	NM_019891	ref|Homo sapiens ERO1-like beta (S. cerevisiae) (ERO1LB), mRNA [NM_019891]	3,150
TMEFF2	AB004064	gb|Homo sapiens mRNA for tomoregulin, complete cds. [AB004064]	3,149
SC5DL	NM_001024956	ref|Homo sapiens sterol-C5-desaturase (ERG3 delta-5-desaturase homolog, S. cerevisiae)-like (SC5DL), transcript variant 2, mR	3,142
PTGS2	NM_000963	ref|Homo sapiens prostaglandin-endoperoxide synthase 2 (prostaglandin G/H synthase and cyclooxygenase) (PTGS2), mRNA [N	3,132
PDGFA	NM_002607	ref|Homo sapiens platelet-derived growth factor alpha polypeptide (PDGFA), transcript variant 1, mRNA [NM_002607]	3,112
HBP1	NM_012257	ref|Homo sapiens HMG-box transcription factor 1 (HBP1), mRNA [NM_012257]	3,110
C17orf59	NM_017622	ref|Homo sapiens chromosome 17 open reading frame 59 (C17orf59), mRNA [NM_017622]	3,105
MXD1	NM_002357	ref|Homo sapiens MAX dimerization protein 1 (MXD1), mRNA [NM_002357]	3,090
SCHIP1	NM_014575	ref|Homo sapiens schwannomin interacting protein 1 (SCHIP1), mRNA [NM_014575]	3,086
AA593970	AA593970	gb|AA593970 nn01c05.s1 NCI_CGAP_Co9 Homo sapiens cDNA clone IMAGE:1076456 3', mRNA sequence [AA593970]	3,073
FASN	NM_004104	ref|Homo sapiens fatty acid synthase (FASN), mRNA [NM_004104]	3,071

GOLGB1	NM_004487	ref|Homo sapiens golgin B1, golgi integral membrane protein (GOLGB1), mRNA [NM_004487]	3,057
TMEM49	BC024020	gb|Homo sapiens transmembrane protein 49, mRNA (cDNA clone IMAGE:4295896), with apparent retained intron. [BC024020]	3,056
PLA2G4C	NM_003706	ref|Homo sapiens phospholipase A2, group IVC (cytosolic, calcium-independent) (PLA2G4C), mRNA [NM_003706]	3,056
ENST00000298876	ENST00000298876	ens|Uncharacterized protein C12orf70 [Source:UniProtKB/Swiss-Prot;Acc:A6NFE2] [ENST00000298876]	3,048
GADD45A	NM_001924	ref|Homo sapiens growth arrest and DNA-damage-inducible, alpha (GADD45A), mRNA [NM_001924]	3,039
C4orf18	NM_016613	ref|Homo sapiens chromosome 4 open reading frame 18 (C4orf18), transcript variant 2, mRNA [NM_016613]	3,032
AMOTL2	NM_016201	ref|Homo sapiens angiomotin like 2 (AMOTL2), mRNA [NM_016201]	3,028
ADAMTS1	NM_006988	ref|Homo sapiens ADAM metallopeptidase with thrombospondin type 1 motif, 1 (ADAMTS1), mRNA [NM_006988]	3,021
CASP4	NM_033306	ref|Homo sapiens caspase 4, apoptosis-related cysteine peptidase (CASP4), transcript variant gamma, mRNA [NM_033306]	3,015
TM7SF2	NM_003273	ref|Homo sapiens transmembrane 7 superfamily member 2 (TM7SF2), mRNA [NM_003273]	3,012
NXN	NM_022463	ref|Homo sapiens nucleoredoxin (NXN), mRNA [NM_022463]	3,009
AK022044	AK022044	gb|Homo sapiens cDNA FLJ11982 fis, clone HEMBB1001335. [AK022044]	3,006
C17orf59	NM_017622	ref|Homo sapiens chromosome 17 open reading frame 59 (C17orf59), mRNA [NM_017622]	2,995
HOXB3	NM_002146	ref|Homo sapiens homeobox B3 (HOXB3), mRNA [NM_002146]	2,976
ABCB1	NM_000927	ref|Homo sapiens ATP-binding cassette, sub-family B (MDR/TAP), member 1 (ABCB1), mRNA [NM_000927]	2,975
ABCB1	NM_000927	ref|Homo sapiens ATP-binding cassette, sub-family B (MDR/TAP), member 1 (ABCB1), mRNA [NM_000927]	2,968
FAM132A	NM_001014980	ref|Homo sapiens family with sequence similarity 132, member A (FAM132A), mRNA [NM_001014980]	2,959
CUL4B	NM_003588	ref|Homo sapiens cullin 4B (CUL4B), transcript variant 1, mRNA [NM_003588]	2,955
GGPS1	NM_004837	ref|Homo sapiens geranylgeranyl diphosphate synthase 1 (GGPS1), transcript variant 1, mRNA [NM_004837]	2,952
ABCB1	NM_000927	ref|Homo sapiens ATP-binding cassette, sub-family B (MDR/TAP), member 1 (ABCB1), mRNA [NM_000927]	2,952
PTGS2	NM_000963	ref|Homo sapiens prostaglandin-endoperoxide synthase 2 (prostaglandin G/H synthase and cyclooxygenase) (PTGS2), mRNA [N	2,944
ERO1LB	NM_019891	ref|Homo sapiens ERO1-like beta (S. cerevisiae) (ERO1LB), mRNA [NM_019891]	2,939
THC2755690	THC2755690	Unknown	2,916
SOCS2	NM_003877	ref|Homo sapiens suppressor of cytokine signaling 2 (SOCS2), mRNA [NM_003877]	2,908
FAM100B	NM_182565	ref|Homo sapiens family with sequence similarity 100, member B (FAM100B), mRNA [NM_182565]	2,904
ANKRD42	NM_182603	ref|Homo sapiens ankyrin repeat domain 42 (ANKRD42), mRNA [NM_182603]	2,902
GADD45B	NM_015675	ref|Homo sapiens growth arrest and DNA-damage-inducible, beta (GADD45B), mRNA [NM_015675]	2,899
LOC100130910	XM_001715202	gb|PREDICTED: Homo sapiens hypothetical protein LOC100130910 (LOC100130910), mRNA [XM_001715202]	2,894
ACLY	U18197	gb|Human ATP:citrate lyase mRNA, complete cds. [U18197]	2,891
MANBAL	NM_022077	ref|Homo sapiens mannosidase, beta A, lysosomal-like (MANBAL), transcript variant 1, mRNA [NM_022077]	2,864
SORBS2	AF090937	gb|Homo sapiens clone HQ0618 PRO0618 mRNA, complete cds. [AF090937]	2,862
CASP4	NM_033306	ref|Homo sapiens caspase 4, apoptosis-related cysteine peptidase (CASP4), transcript variant gamma, mRNA [NM_033306]	2,852
HCG18	NR_024052	ref|Homo sapiens HLA complex group 18 (HCG18), non-coding RNA [NR_024052]	2,850
ABCB1	NM_000927	ref|Homo sapiens ATP-binding cassette, sub-family B (MDR/TAP), member 1 (ABCB1), mRNA [NM_000927]	2,834
KIAA1875	NR_024207	ref|Homo sapiens KIAA1875 (KIAA1875), non-coding RNA [NR_024207]	2,829
TXK	NM_003328	ref|Homo sapiens TXK tyrosine kinase (TXK), mRNA [NM_003328]	2,818
ENST00000379619	ENST00000379619	ens|Fos-related antigen 2 [Source:UniProtKB/Swiss-Prot;Acc:P15408] [ENST00000379619]	2,809
GOLGB1	X75304	gb|H.sapiens giantin mRNA. [X75304]	2,808
TMCO3	NM_017905	ref|Homo sapiens transmembrane and coiled-coil domains 3 (TMCO3), mRNA [NM_017905]	2,802
THC2682560	THC2682560	Unknown	2,801
PGD	NM_002631	ref|Homo sapiens phosphogluconate dehydrogenase (PGD), mRNA [NM_002631]	2,800
THC2739352	THC2739352	thc|Q3GZE6_9ACTO (Q3GZE6) AAA ATPase, central region, partial (3%) [THC2739352]	2,793
HACL1	NM_012260	ref|Homo sapiens 2-hydroxyacyl-CoA lyase 1 (HACL1), mRNA [NM_012260]	2,782
C5orf26	NR_015370	ref|Homo sapiens chromosome 5 open reading frame 26 (C5orf26), non-coding RNA [NR_015370]	2,777
MC5R	NM_005913	ref|Homo sapiens melanocortin 5 receptor (MC5R), mRNA [NM_005913]	2,776
ERICH1	NM_207332	ref|Homo sapiens glutamate-rich 1 (ERICH1), mRNA [NM_207332]	2,759
PRDM10	NM_199437	ref|Homo sapiens PR domain containing 10 (PRDM10), transcript variant 2, mRNA [NM_199437]	2,754
KLC1	NM_182923	ref|Homo sapiens kinesin light chain 1 (KLC1), transcript variant 2, mRNA [NM_182923]	2,749
ABCB1	NM_000927	ref|Homo sapiens ATP-binding cassette, sub-family B (MDR/TAP), member 1 (ABCB1), mRNA [NM_000927]	2,745
LSS	NM_002340	ref|Homo sapiens lanosterol synthase (2,3-oxidosqualene-lanosterol cyclase) (LSS), transcript variant 1, mRNA [NM_002340]	2,729
RP4-691N24.1	NM_025176	ref|Homo sapiens ninein-like (NLP), mRNA [NM_025176]	2,718
ABCB1	NM_000927	ref|Homo sapiens ATP-binding cassette, sub-family B (MDR/TAP), member 1 (ABCB1), mRNA [NM_000927]	2,706
JUND	X56681	gb|Human junD mRNA. [X56681]	2,693
SKAP1	NM_003726	ref|Homo sapiens src kinase associated phosphoprotein 1 (SKAP1), transcript variant 1, mRNA [NM_003726]	2,681
AUH	NM_001698	ref|Homo sapiens AU RNA binding protein/enoyl-Coenzyme A hydratase (AUH), nuclear gene encoding mitochondrial protein, m	2,666
ANKRD11	NM_013275	ref|Homo sapiens ankyrin repeat domain 11 (ANKRD11), mRNA [NM_013275]	2,666
CELSR3	NM_001407	ref|Homo sapiens cadherin, EGF LAG seven-pass G-type receptor 3 (flamingo homolog, Drosophila) (CELSR3), mRNA [NM_00	2,656
LRRIQ1	NM_032165	ref|Homo sapiens leucine-rich repeats and IQ motif containing 1 (LRRIQ1), transcript variant 2, mRNA [NM_032165]	2,639
THC2656875	THC2656875	Unknown	2,638
DEPDC6	NM_022783	ref|Homo sapiens DEP domain containing 6 (DEPDC6), mRNA [NM_022783]	2,620
PNPLA3	NM_025225	ref|Homo sapiens patatin-like phospholipase domain containing 3 (PNPLA3), mRNA [NM_025225]	2,581
LOC92270	NM_001017971	ref|Homo sapiens V-type proton ATPase subunit S1-like protein (LOC92270), mRNA [NM_001017971]	2,579
RASSF1	NM_170713	ref|Homo sapiens Ras association (RalGDS/AF-6) domain family member 1 (RASSF1), transcript variant C, mRNA [NM_170713	2,574
LOC153546	AK055939	gb|Homo sapiens cDNA FLJ31377 fis, clone NESOP1000087. [AK055939]	2,574
ANKRD42	NM_182603	ref|Homo sapiens ankyrin repeat domain 42 (ANKRD42), mRNA [NM_182603]	2,559
BX100171	BX100171	gb|BX100171 Soares_testis_NHT Homo sapiens cDNA clone IMAGp998C011795, mRNA sequence [BX100171]	2,529
